# Peritoneal neutrophil extracellular traps contribute to septic AKI via peritoneal IL-17A and distant organ CXCL-1/ CXCL-2 pathway in abdominal sepsis

**DOI:** 10.1038/s41598-025-34770-1

**Published:** 2026-01-27

**Authors:** Yoshitaka Naito, Daiki Goto, Naoki Hayase, Xuzhen Hu, Peter S. T. Yuen, Robert A. Star

**Affiliations:** Renal Diagnostics and Therapeutics Unit, National Institute of Diabetes and Digestive and Kidney Diseases (NIDDK), NIH, Building 10, Room 9N230, 10 Center Drive, MSC 1268, Bethesda, MD 20892-1268 USA

**Keywords:** Diseases, Immunology, Medical research, Nephrology

## Abstract

There are no specific treatments for Sepsis-associated acute kidney injury (AKI). We previously reported that *Il-17a*-knockout mice had dramatically improved survival after cecal ligation and puncture (CLP). Neutrophil extracellular traps (NETs) induce IL-17A, which causes harm in some diseases, but this pathway is poorly understood in sepsis. We found that knockout of *Pad4* (Peptidyl Arginine Deiminase 4), an enzyme essential for NET formation, improved survival and AKI, and suppressed neutrophil infiltration into remote organs, involving a peritoneal IL-17A/distant organ CXCL-1/CXCL-2 pathway after CLP. NETs were detected in the peritoneal cavity, and not in plasma or distant organs. Adoptive transfer of peritoneal WT neutrophils restored the IL-17A/CXCL-1/CXCL-2 pathway in *Pad4*KO mice, leading to neutrophil infiltration and damage to remote organs. These results revealed a pathway from peritoneal NET formation to remote organ injury/inflammation via production of IL-17A at the infectious site and distant organ CXCL-1/CXCL-2. While NETs promoted intraperitoneal IL-17A production, we also showed that conversely, peritoneal IL-17A or CXCL-1/CXCL-2 promoted intraperitoneal NET formation after CLP. This peritoneal vicious cycle that includes NET formation, IL-17A, CXCL-1/CXCL-2 that may amplify sepsis-associated organ injury. Breaking this vicious cycle by inhibiting NET formation and/or IL-17A might be a promising therapeutic target for sepsis treatment.

**Supplementary Information:**

The online version contains supplementary material available at 10.1038/s41598-025-34770-1.

## Introduction

About 60% of septic patients develop sepsis-associated acute kidney injury (AKI)^1–3^, with a mortality rate of 60–70%^1,4^. Multiple organ failure is a central feature of severe sepsis and septic shock, including kidneys and lungs^5–7^. There are no specific treatments for sepsis-associated AKI and lung injury^8^. Neutrophil extracellular traps (NETs), expelled from activated neutrophils, contain cell-free DNA (cfDNA), histones, chromatin, proteases, and antimicrobial proteins, and may be the primary source of cfDNA in early sepsis^9–13^. Peptidylarginine deiminase 4 (PAD4) is essential for NET formation through histone citrullination^14^, thereby inducing chromatin decondensation^15^. Protein kinase Cα and cyclin-dependent kinase 4/6 can mediate nuclear envelope rupture via lamin phosphorylation^16,17^. These processes can induce cell membrane disintegration associated with NETosis, accompanied by cytoskeletal remodeling^18^. NETs have dual effects in sepsis. They can immobilize bacteria, which is advantageous; but their components (histones, chromatin, proteases, and cfDNA) can harm host cells^10,11,13^. Due to these opposing effects, targeting NETs in sepsis may be difficult. Martinod et al. observed that *Pad4* knockout did not enhance survival in a mild CLP model and worsened survival in a severe CLP model^19^. Others have shown that *Pad4* knockout or DNase treatment improved organ damage in a mouse model of *E. coli* sepsis^20–22^. Moreover, blocking NETs with a non-specific PAD inhibitor Cl-amidine, antibodies against citrullinated histone H3 (H3Cit), or DNase enhanced survival post-CLP^23,24^. These apparently contradictory effects imply that NETs exert both beneficial and detrimental effects in sepsis, contingent upon the particular context.

Interestingly, NETs increase IL-17A, a cytokine we previously demonstrated to be downstream of TLR9^25^, a known mediator of sepsis^26,27^. NETs promote Th17 cell differentiation through one of their components, histone^28^. *Pad4* knockout halted IL-17A accumulation in atherosclerosis within the aorta^29^. However, the contribution of NET formation to IL-17A production in sepsis remains unclear.

We hypothesized that NETs increase IL-17A and its downstream factors, CXCL-1 and CXCL-2, causing neutrophil infiltration and organ injury in sepsis. Using two knock out mouse models (*Pad4*KO mice in which NET formation was completely abolished; and *Il-17a*KO), we evaluated the 1) effect of NET formation on CLP outcomes; 2) main sites of NET formation in CLP-treated mice; 3) effect of NET formation on neutrophil infiltration into remote organs and IL-17A and CXCL-1/CXCL-2 pathway after CLP; 4) relationship between NET formation and IL-17A in the peritoneal cavity; and 5) effect of peritoneal NETs on remote organ injury using an adoptive transfer model of wild type (WT) neutrophils into *Pad4*KO mice after CLP.

## Results

### Effect of knockout of *pad4* on survival, AKI, and neutrophil infiltration into kidney and lung after CLP

**Fig. 1:**
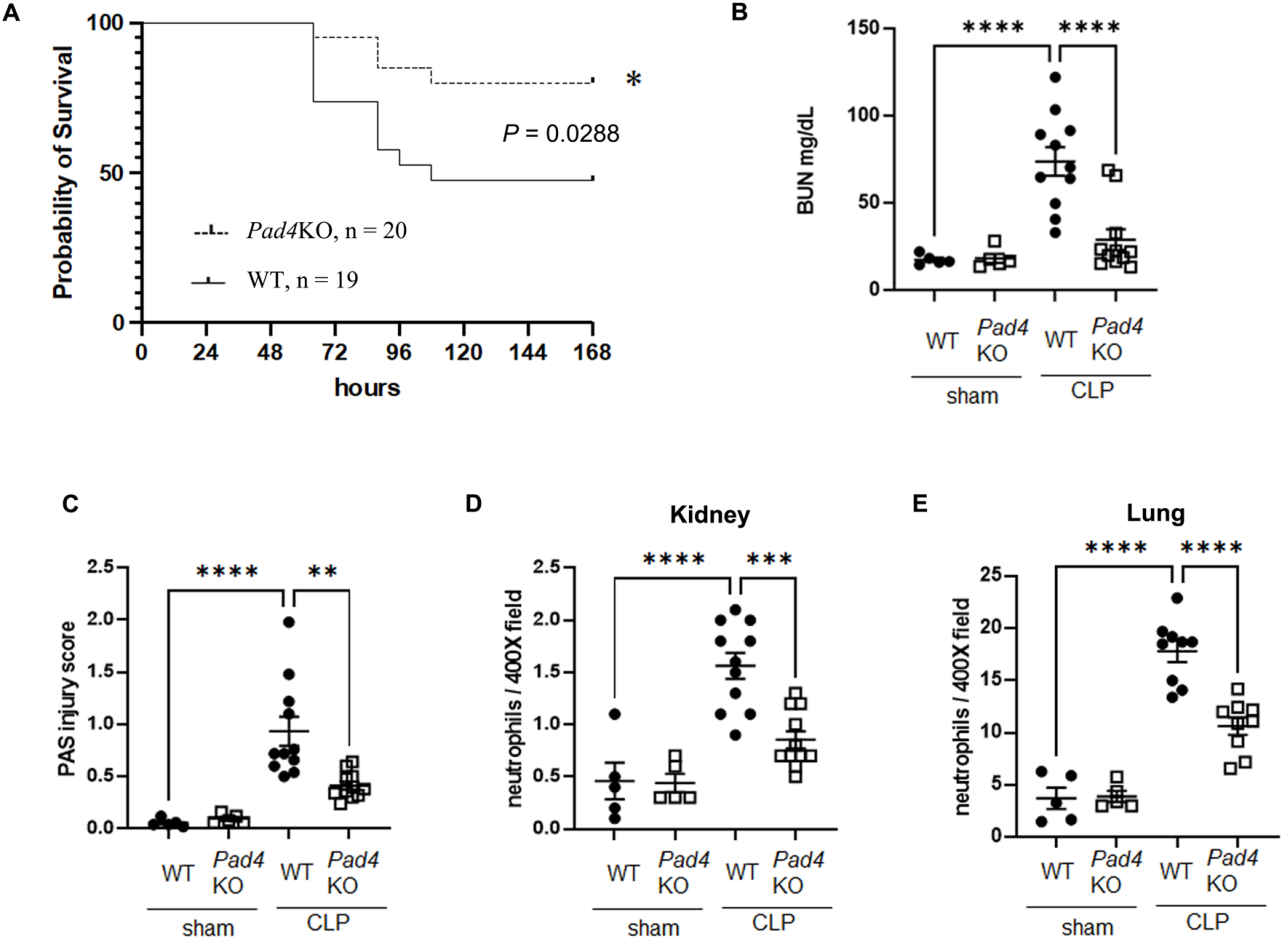
Knockout of *Pad4* improved survival, AKI, and neutrophil infiltration into kidney and lung after cecal ligation and puncture (CLP). (**A**) Survival curves of wild-type (WT) mice (n = 19) or *Pad4* knockout (*Pad4*KO) mice (n = 20) after CLP surgery. The survival data were analyzed by a log-rank test. *P < 0.05 vs. WT mice. (**B**) Plasma BUN levels in WT or *Pad4*KO mice at 18 h after sham (n = 5 per group) or CLP (n = 11 per group) surgery. (**C**) Tubular damage score in kidney cortex of WT or *Pad4*KO mice at 18 h after sham (n = 5 per group) or CLP surgery (n = 11 per group). (**D** and **E**) Number of neutrophils in kidney (**D**) and lung (**E**) of WT or *Pad4*KO mice at 18 h after sham (n = 5 per group) or CLP surgery (n = 11 per group) using naphthol AS-D chloroacetate esterase staining. The data sets were analyzed by one-way ANOVA, followed by Tukey’s multiple comparisons test. Values represent the means ± SEM. **P < 0.01, *** *P* < 0.001, **** *P* < 0.0001.

To investigate the role of NET formation in our moderate severity, clinically relevant model of polymicrobial sepsis, we examined whether knockout of *Pad4* alters survival, renal function and morphology, and neutrophil infiltration into kidney and lung after CLP. *Pad4KO* mice had significantly improved survival at 168 h after CLP, with a survival rate of 80% compared to 47% for WT mice (Fig. 1A). WT mice developed kidney injury 18 h after CLP, whereas *Pad4*KO mice showed decreased serum blood urea nitrogen (BUN) levels (Fig. 1B) and improved cortical tubular damage scores (Fig. 1C and Supplemental Fig. 1). Neutrophil infiltration into kidney and lung were increased at 18 h after CLP, (Fig. 1D and E,, and Supplemental Fig. 2A and B), but *Pad4*KO mice exhibited significantly lower neutrophil infiltration. Knockout of *Pad4* did not alter the number of bacterial colonies 18 h after CLP (Supplemental Fig. 3A).

### Effect of knockout of *Pad*4 on CXCL-1 and -2 production in kidney and lung, and IL-17A production in peritoneal lavage fluid (PLF) and plasma after CLP

**Fig. 2:**
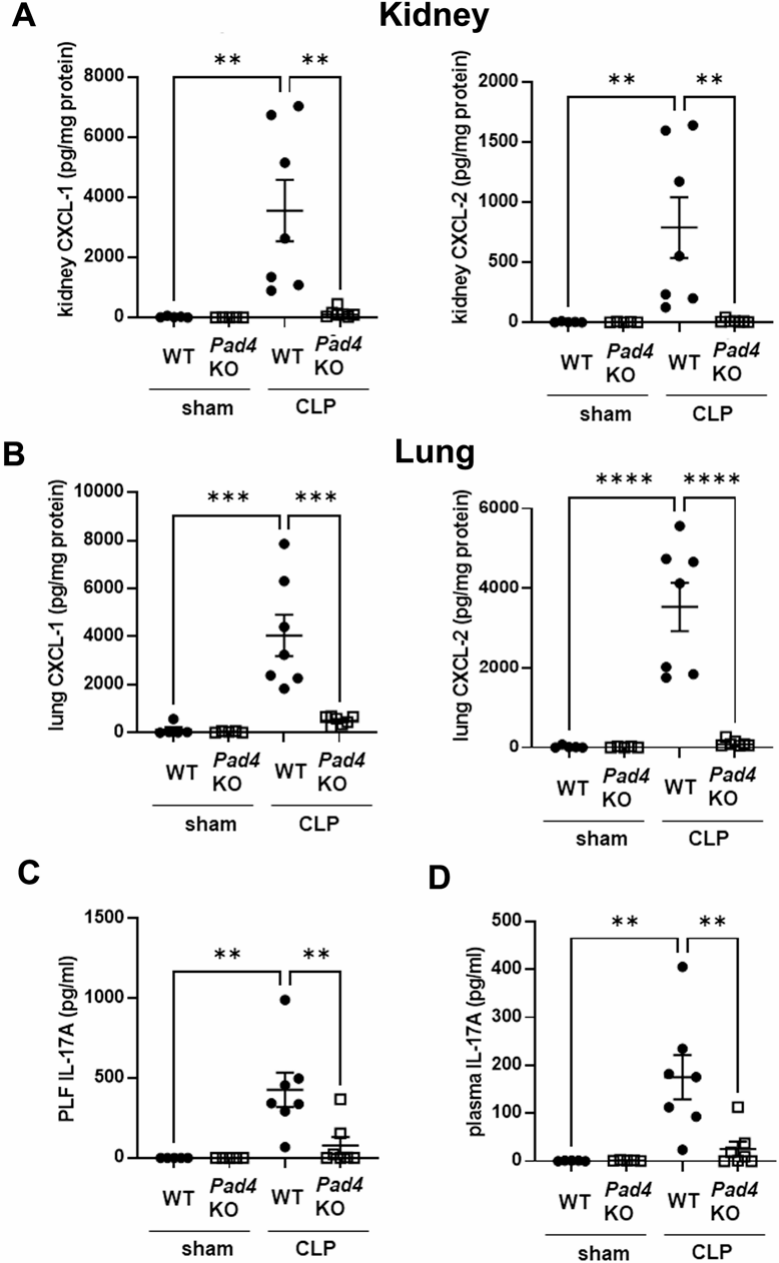
Knockout of *Pad4* inhibited CXCL-1 and -2 production in kidney and lung and IL-17A production in peritoneal cavity and plasma at 18 h after CLP. (A-B) CXCL-1 and -2 concentration in kidney (**A**) and lung (**B**) of WT or *Pad4*KO mice at18 h after sham (n = 5 per group) or CLP (n = 7 per group) surgery. (**C**, **D**) IL-17A concentration in PLF (**C**) and plasma (**D**) at 18 h after sham (n = 5 per group) or CLP surgery (n = 7 per group). The data sets were analyzed by one-way ANOVA, followed by Tukey’s multiple comparisons test. Values represent the means ± SEM. **P < 0.01, *** *P* < 0.001, **** *P* < 0.0001.

To investigate the role of NET formation in CXCL-1 and -2 production in kidney and lung after CLP, CXCL-1 and -2 levels in kidney and lung were evaluated in *Pad4*KO and WT mice 18 h after sham or CLP surgery (Fig. 2A and B). CXCL-1 and -2 levels were significantly higher in kidney and lung from WT mice vs. *Pad4*KO mice at 18 h after CLP. Levels of IL-17A, a known upstream factor of CXCL-1 and -2^30^, in PLF and plasma were also assessed after CLP (Fig. 2C and D). IL-17A levels were significantly higher in PLF and plasma of WT mice vs. *Pad4*KO mice 18 h after CLP.

### Effect of knockout of *Il-17a* on AKI, neutrophil infiltration into kidney and lung, and levels of CXCL-1 and -2 in kidney and lung after CLP

**Fig. 3:**
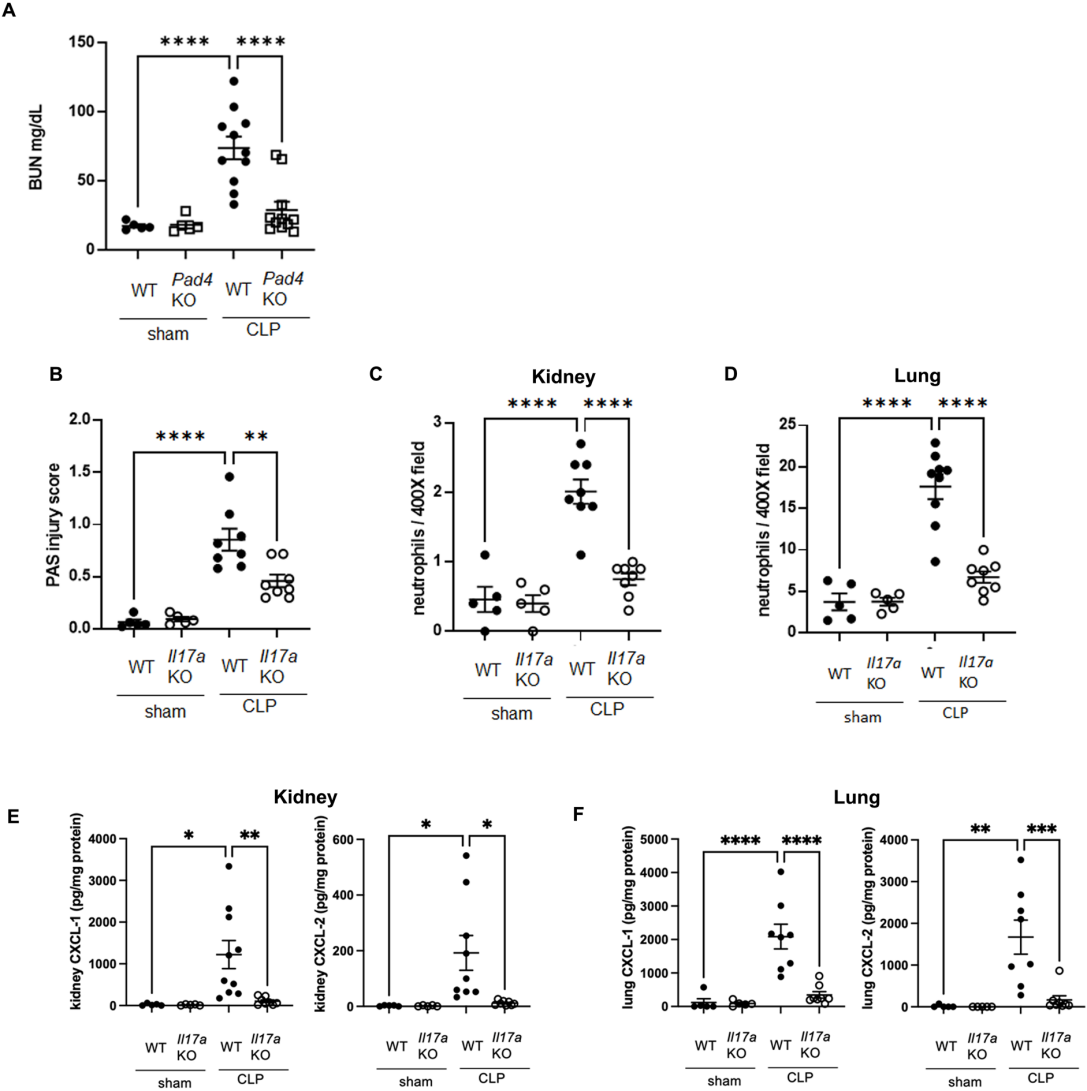
Knockout of *Il-17a* attenuated AKI and neutrophil infiltration into kidney and lung, and inhibited CXCL-1 and -2 production in kidney and lung at 18 h after CLP. (**A**) Plasma BUN levels in WT or *Il-17a*KO mice at 18 h after sham (n = 6 per group) or CLP (n = 10 per group) surgery. (**B**) Tubular damage score in kidney cortex of WT or *Il-17a*KO mice at 18 h after sham (n = 5 per group) or CLP surgery (n = 8 per group). (**C**) Number of neutrophils in kidney of WT or *Il-17a*KO mice at 18 h after sham (n = 5 per group) or CLP surgery (n = 8 per group) using naphthol AS-D chloroacetate esterase staining. Neutrophils were counted in × 400 fields and averaged per mouse. (**D**) Number of neutrophils in lung of WT or *Il-17a*KO mice at 18 h after sham (n = 5 per group) or CLP surgery (n = 9 per group) using naphthol AS-D chloroacetate esterase staining. Neutrophils were counted in × 400 fields and averaged per mouse. (**E**, **F**) CXCL-1 and -2 concentration in kidney (**E**) and lung (**F**) of WT or *Il-17a*KO mice at18 h after sham (n = 5 per group) or CLP (n = 8–10 per group) surgery. The data sets were analyzed by one-way ANOVA, followed by Tukey’s multiple comparisons test. Values represent the means ± SEM. *P < 0.05, **P < 0.01, *** *P* < 0.001, **** *P* < 0.0001.

*Il-17a* KO improves survival in sepsis^25^, but the mechanism is unknown. To investigate the role of IL-17A in sepsis-associated AKI, plasma BUN levels (Fig. 3A) and renal tubular damage scores (Fig. 3B and Supplemental Fig. 4) were assessed 18 h after CLP. WT mice developed kidney injury 18 h after CLP, whereas *Il-17a*KO mice showed decreased BUN levels (Fig. 3A) and improved tubular damage scores in the cortex (Fig. 3B and Supplemental Fig. 4) similar to *Pad4*KO mice. Knockout of *Il-17a* significantly decreased neutrophil infiltration into kidney and lung at 18 h after CLP compared with WT mice by naphthol AS-D chloroacetate esterase staining (Fig. 3C and D, and Supplemental Fig. 5A and B). Neutrophil infiltration into kidney was also assessed by flow cytometry (Supplemental Fig. 6A, B, C, and D). Neutrophil infiltration was upregulated by CLP at 3 and 18 h after CLP, which was attenuated in *Il-17a* KO mice. Furthermore, knockout of *Il-17a*, similar to knockout of *Pad4,* significantly decreased CXCL-1 and -2 levels in kidney and in lung at 18 h after CLP compared to WT mice (Fig. 3E and F). Knockout of *Il-17a* did not alter the number of bacterial colonies in PLF collected at 18 h after CLP (Supplemental Fig. 3B).

### NET formation in peritoneal cavity after CLP

**Fig. 4:**
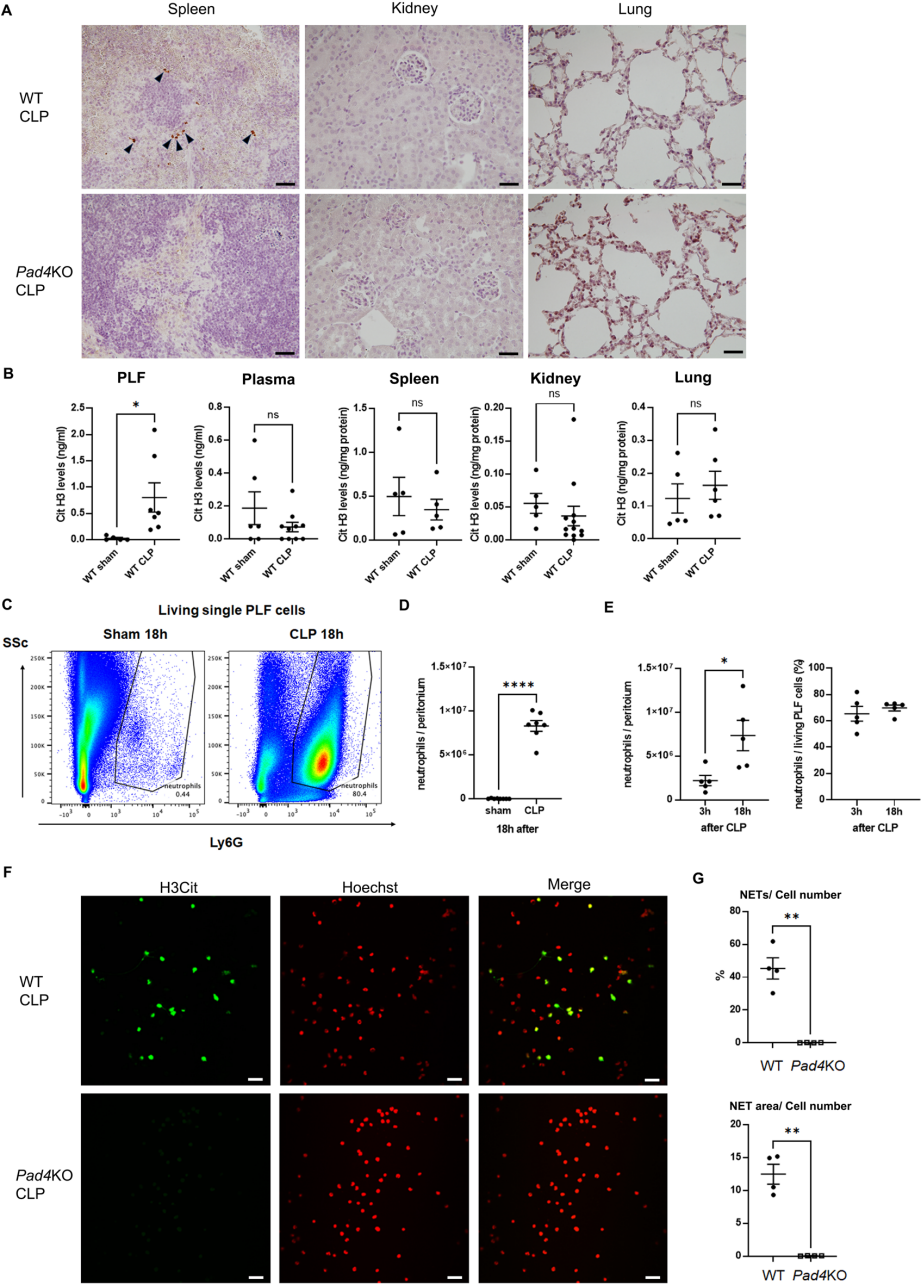
NET formation was detected only in the peritoneal cavity, and not in plasma, spleen, kidney, or lung at 18 h after CLP. (**A**) H3Cit staining in spleen, kidney, and lung of WT or *Pad4*KO mice at 18 h after CLP. Original magnification, × 400. (**B**) H3Cit levels in PLF, plasma, spleen, kidney, and lung at at 18 h after sham (n = 5–6 per group) or CLP (n = 5–12 per group) surgery. (**C**) Representative images of flow cytometry analysis for Ly6G + neutrophils in PLF cells at 18 h after sham or CLP surgery. Living (zombie violet negative), CD45 + cells were gated from singlets. Then Ly6G expression was analyzed in this population. (**D**) Ly6G + neutrophil population in peritoneal cavity at 18 h after sham or CLP surgery (n = 7–8 per group). (**E**) The course of absolute number (right) or percentage (left) of Ly6G + , CD11b + neutrophil accumulation in peritoneal cavity after CLP surgery (3 and 18 h). Living cells (7-AAD negative) were gated from singlets. Then Ly6G and CD11b positive cells were gated and counted as neutrophils (n = 5 per group). (**F**) Representative images of NET formation in PLF cells of WT or *Pad4*KO mice at 18 h after CLP. PLF cells were placed on poly-L-lysine coated cover slips without additional stimulation and incubated at 37 °C for 2 h. These cells were stained for H3Cit (Green) or Hoechst (Red Pseudo color). Original magnification, × 400. (**G**) Summary analysis of NET formation in PLF cells of WT (n = 5) or *Pad4*KO mice (n = 4) at 18 h after CLP. Percentage of cells with NET formation was calculated as the number of cells extruding H3Cit-positive structures divided by the number of cells identified by Hoechst and multiplied by 100 (upper). NET extension in PLF cells was also measured as the H3Cit-positive area divided by the number of cells evaluated by Hoechst (lower). The data sets were analyzed by unpaired t-test. Values represent the means ± SEM. ns: not significant, *P < 0.05, **P < 0.01, **** *P* < 0.0001. Scale bars = 20 µm.

We next investigated where NETs can form in CLP-treated mice. Spleen, kidney, and lung were harvested from WT or *Pad4*KO mice 18 h after CLP and stained for H3Cit (Fig. 4A). Few H3Cit-positive cells were observed in a very limited area in the spleen, and not at all in kidney and lung. H3Cit levels significantly increased in CLP treated mice compared to sham only in PLF, but not in plasma, spleen, or kidney (Fig. 4B). Since evidence of NET formation was detected only in the PLF in our CLP model, we focused on neutrophils in the peritoneal cavity. CLP significantly increased neutrophil accumulation in PLF at 18 h after CLP (Fig. 4D). Also, among live PLF cells the percentage of neutrophils was as high as 65.36% (95%CI 51.27–83.30%) at 3 h and 69.78% (95%CI 63.38–76.86%) 18 h after CLP, even without neutrophil purification (Fig. 4E). Approximately 40% of PLF cells from WT mice formed NETs after CLP without any ex vivo stimulation (Fig. 4F), and NETs were almost completely absent in cells from *Pad4KO* mice (Fig. 4G). A similar trend was obtained when the H3Cit-positive area was normalized by cell number, which we interpreted as NET extension in PLF cells (Fig. 4G).

### The effect of knockout of *Il-17a* on CXCL-1 and -2 production in PLF and plasma and NET formation in peritoneal cavity

**Fig. 5:**
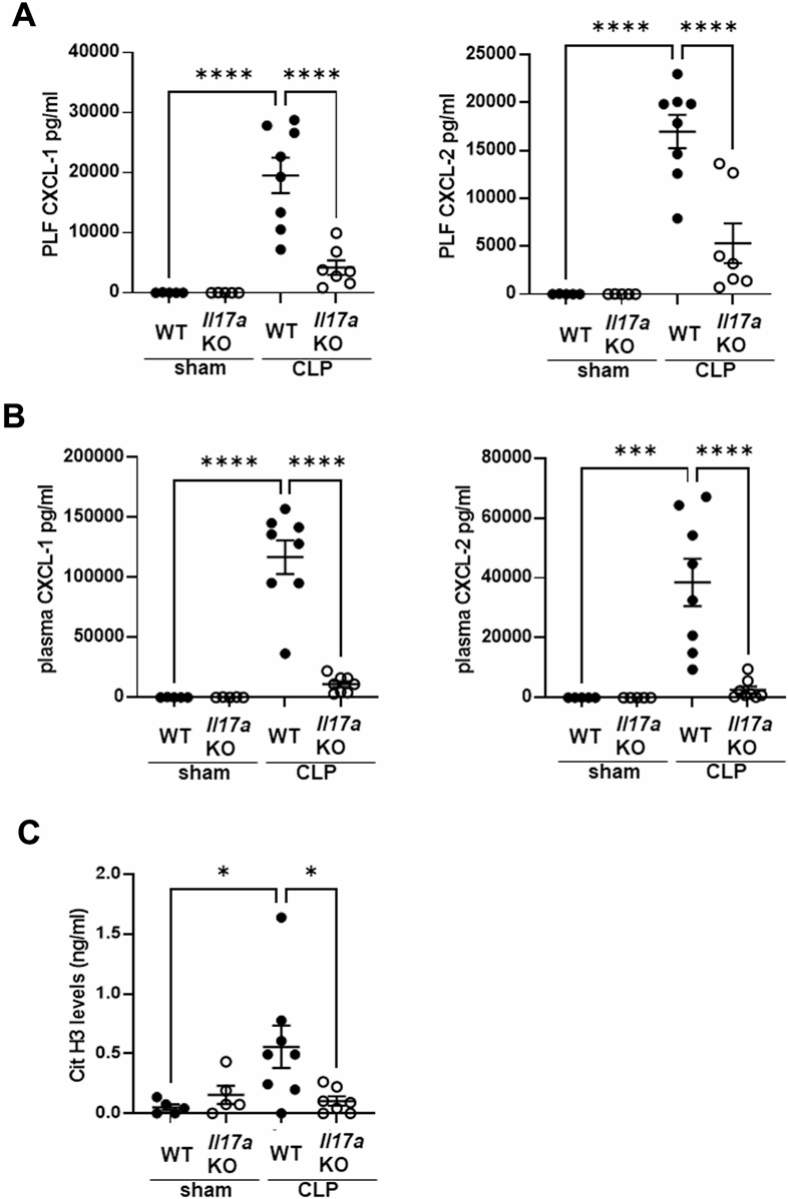
Knockout of *Il-17a* suppressed CXCL-1 and -2 content in PLF and plasma and H3Cit levels in PLF at 18 h after CLP. (**A**, **B**) CXCL-1 and -2 concentration in PLF (**A**) and plasma (**B**) of WT or *Il-17a*KO mice at 18 h after sham (n = 5) or CLP (n = 8) surgery. (**C**) H3Cit levels in PLF of WT or *Il-17a*KO mice at 18 h after sham (n = 5) or CLP (n = 7–8) surgery. The data sets were analyzed by one-way ANOVA, followed by Tukey’s multiple comparisons test. Values represent the means ± SEM. *P < 0.05, ***P < 0.001, ****P < 0.0001.

In PLF and plasma, CXCL-1 and -2 levels were upregulated 18 h after CLP in WT mice, whereas these levels were significantly decreased in *Il-17a*KO mice (Fig. 5A and B). Knockout of *Il-17a* did not significantly alter the absolute number or percentage of neutrophils infiltrating into the peritoneal cavity 18 h after CLP (Supplemental Fig. 7). However, H3Cit levels in PLF, which were elevated 18 h after CLP in WT mice, were significantly decreased in PLF from *Il-17a* KO mice (Fig. 5C).

### The effect of IL-17A, CXCL-1, and -2 on NET formation in PLF cells ex vivo

**Fig. 6:**
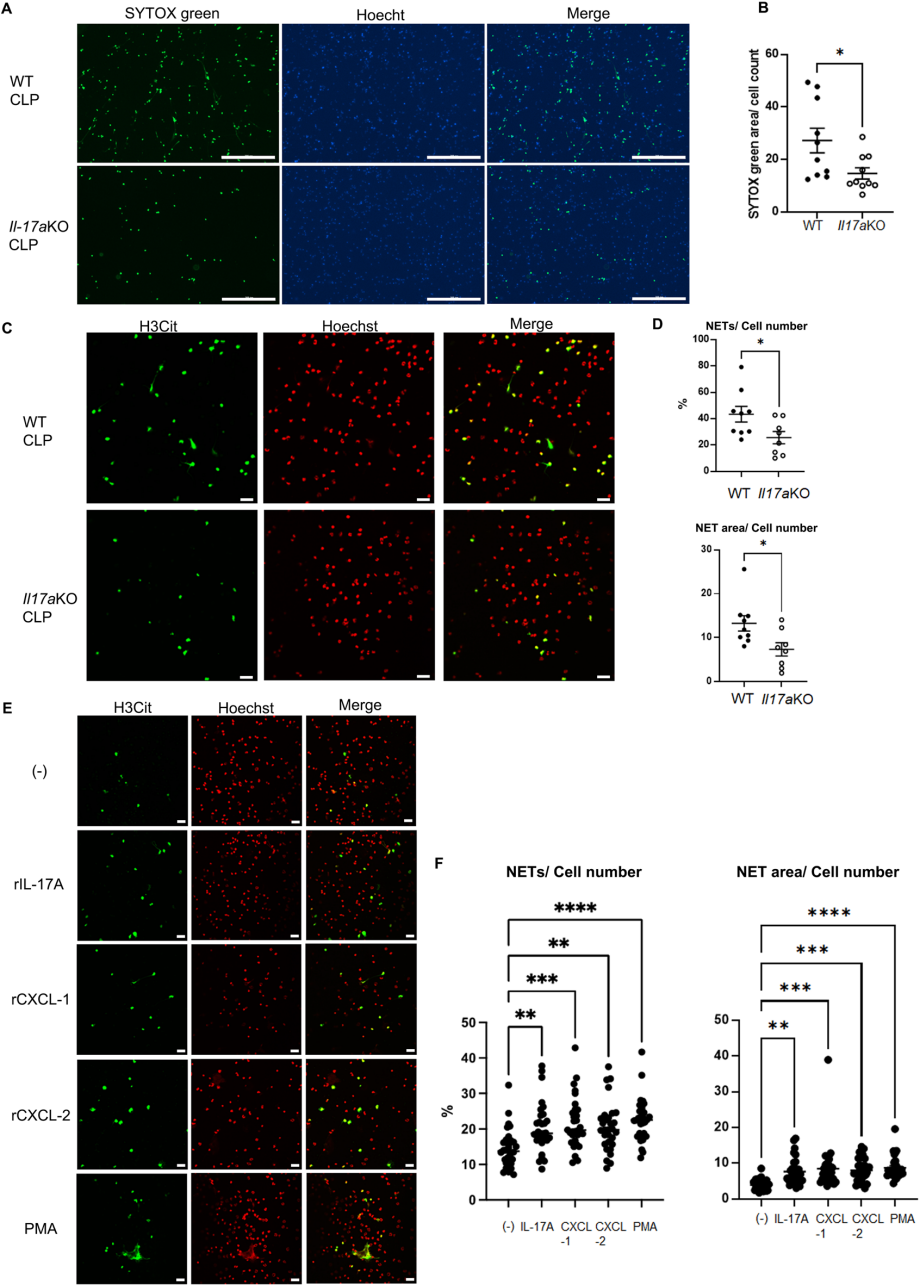
Knockout of *Il-17a* suppressed NET formation in PLF cells ex vivo at 18 h after CLP. Ex vivo stimulation with recombinant IL-17A, CXCL-1, or -2 promoted NET formation in PLF cells. (**A**) Representative images of PLF cells from WT or *Il-17a*KO mice at 18 h after CLP. PLF cells were applied to a 24 well plate without additional stimulation and incubated at 37 °C for 2 h. These cells were stained with SYTOX green (green) and Hoechst (blue). (**B**) NET extension in PLF cells from WT (n = 10) or *Il-17a*KO (n = 10) was measured as the SYTOX green-positive area divided by the number of cells detected by Hoechst. (**C**) Representative images of NET formation in PLF cells from WT or *Il-17a*KO mice at 18 h after CLP. PLF cells were placed on poly-L-lysine coated cover slips without additional stimulation and incubated at 37 °C for 3 h. These cells were stained for H3Cit (Green) and Hoechst (Red pseudocolor). Original magnification, × 400. (**D**) Summary analysis of NET formation in PLF cells of WT (n = 9) or *Il-17a*KO mice (n = 8) at 18 h after CLP. Percentage of cells with NET formation (upper) and NET extension (lower) in PLF cells were calculated. (**E**) Representative images of NET formation in PLF cells from WT mice at 3 h after CLP. PLF cells were stimulated with recombinant IL-17A, CXCL-1, or -2 at 37 °C for 3 h. These cells were stained for H3Cit (Green) and Hoechst (Red pseudocolor). Original magnification, × 400. (**F**) Summary analysis of NET formation in PLF cells collected 3 h after CLP stimulated with recombinant IL-17A, CXCL-1, -2, or PMA for 3 h (30 fields from 2–3 coverslips per group). Percentage of cells with NET formation (left) and NET extension (right) in PLF cells were calculated. The data sets were analyzed by unpaired t-test (**B** and **D**) or one-way ANOVA, followed by Tukey’s multiple comparisons test (**F**). Values represent the means ± SEM. *P < 0.05, **P < 0.01, *** *P* < 0.001, **** *P* < 0.0001. Scale bars = 300 µm (**A**) and 20 µm (**C** and **E**).

PLF cells from *Il-17a KO* mice significantly decreased NET extension, measured as SYTOX green positive area normalized to cell number, compared to WT mice (Fig. 6A and B). NET formation was also assessed by staining with H3Cit antibody using confocal microscopy; PLF cells from *Il-17a*KO mice vs cells from WT mice had a decreased percentage of NET formation or NET extension (Fig. 6C, and D). Next, we evaluated the effects of recombinant IL-17A, CXCL-1, or -2 stimulation on NET formation in PLF cells. Ex vivo incubation of PLF cells collected 3 h after CLP with recombinant IL-17A, CXCL-1, or -2 increased the percentage of NET formation or NET extension (Fig. 6E and F).

### Effect of intraperitoneal adoptive transfer of WT neutrophils into *Pad4* KO mice on septic AKI, lung inflammation, and IL-17A/CXCL-1/CXCL-2 axis

**Fig. 7:**
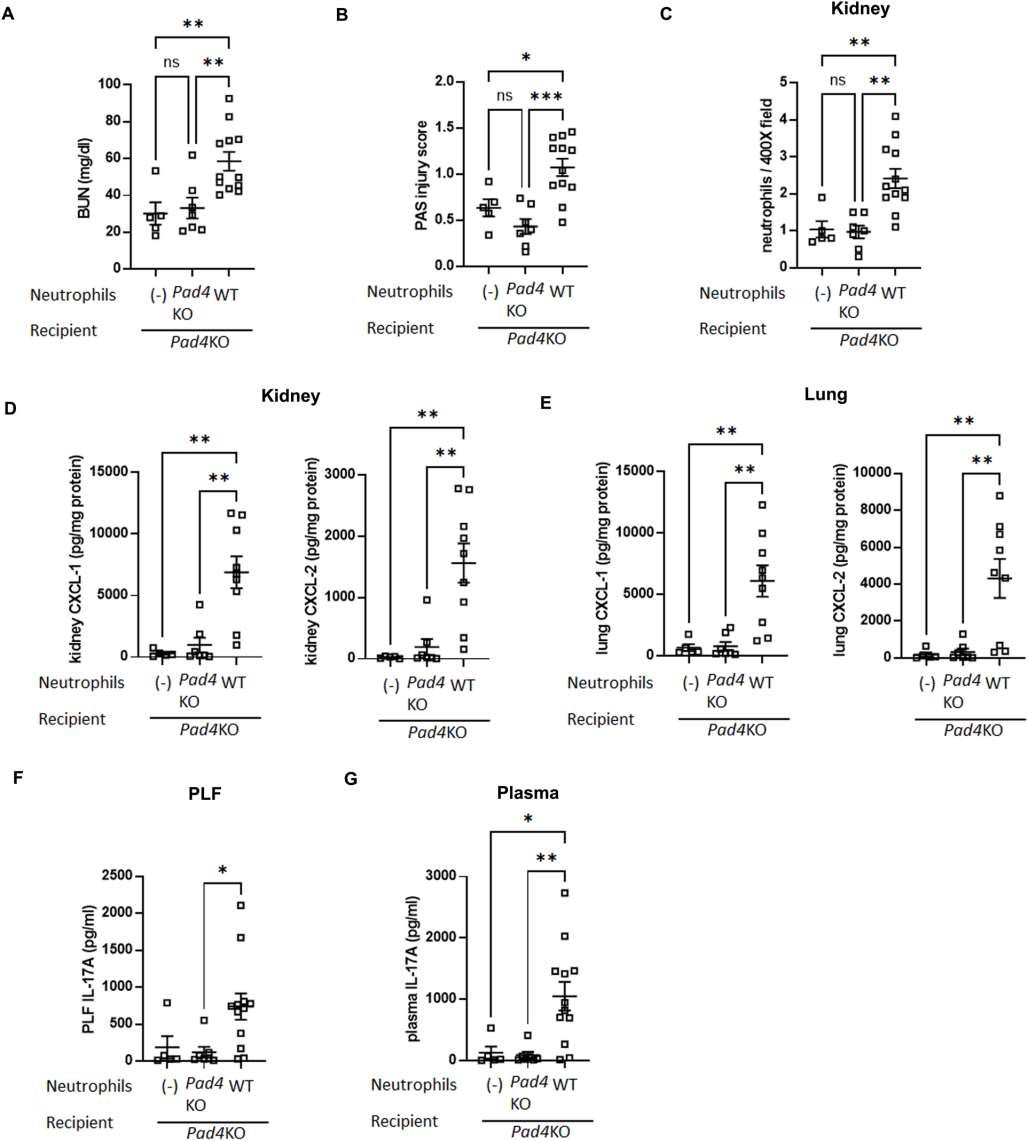
Intraperitoneal adoptive transfer of WT neutrophils counteracted the attenuation of septic AKI, neutrophil infiltration into kidney, IL-17A production in PLF and plasma, CXCL-1/CXCL-2 production in kidney and lung caused by knockout of *Pad4*. (**A**) Plasma BUN levels in *Pad4*KO mice at 18 h after CLP surgery injected with WT (n = 12) or *Pad4*KO (n = 7) neutrophils, or vehicle (n = 5). (**B**) Tubular damage score in kidney cortex in *Pad4*KO mice at 18 h after CLP surgery injected with WT (n = 12) or *Pad4*KO (n = 7) neutrophils, or vehicle (n = 5). (**C**) Number of neutrophils in kidney of *Pad4*KO mice at18 h after CLP surgery injected with WT (n = 12) or *Pad4*KO (n = 7) neutrophils, or vehicle (n = 5) using naphthol AS-D chloroacetate esterase staining. Neutrophils were counted in × 400 fields and averaged per mouse. (**D**, **E**) CXCL-1 and -2 concentration in kidney (**D**) and lung (**E**) of *Pad4*KO mice at 18 h after CLP surgery injected with WT (n = 9) or *Pad4*KO (n = 7) neutrophils, or vehicle (n = 5). (**F**, **G**) IL-17A concentration in (**F**) PLF and (**G**) plasma of *Pad4*KO mice at 18 h after CLP surgery injected with WT (n = 12) or *Pad4*KO (n = 7) neutrophils, or vehicle (n = 5). The data sets were analyzed by one-way ANOVA, followed by Tukey’s multiple comparisons test. Values represent the means ± SEM. ns: not significant, *P < 0.05, **P < 0.01, *** *P* < 0.001. Scale bars = 20 µm.

For further investigation of the relationship between intraperitoneal NET formation and distant organ injuries, we assessed whether adoptive transfer of WT neutrophils into *Pad4*KO mice can reverse the CLP-induced AKI or lung inflammation attenuated by *Pad4* knockout. First, donor WT or *Pad4*KO mice were subjected to CLP. PLF cells were collected at 18 h after CLP and neutrophils were purified from these PLF cells. Then, recipient *Pad4*KO mice were subjected to CLP and neutrophils collected from WT or *Pad4*KO donor mice were intraperitoneally administered into *Pad4*KO mice immediately after CLP of recipients (Supplemental Fig. 8A). In CLP-treated *Pad4*KO mice, WT donor neutrophil administration reconstituted AKI, in contrast with *Pad4*KO donor neutrophil administration or vehicle injection (Fig. 7A and B, and Supplemental Fig. 9A). WT neutrophil administration into *Pad4*KO mice also increased neutrophil infiltration into kidney and lung compared with injection of *Pad4*KO neutrophils or vehicle (Fig. 7C, and Supplemental Fig. 9B, C, and D). We then assessed CXCL-1 and -2 levels in kidney (Fig. 7D) and lung (Fig. 7E) and IL-17A levels in PLF (Fig. 7F) and plasma (Fig. 7G) at 18 h after CLP. Adoptive transfer of WT neutrophils counteracted the attenuation of CXCL-1 and -2 production in kidney and lung and IL-17A production in PLF and plasma by *Pad4* knockout, whereas adoptive transfer of *Pad4*KO neutrophils did not alter these levels. These findings indicate that the beneficial effect on sepsis-associated AKI and lung inflammation induced by *Pad4* knockout are attenuated by intraperitoneal administration of WT neutrophils.

## Discussion

Neutrophils can have both beneficial and harmful effects in sepsis, potentially explaining why neutrophil depletion does not change overall survival in CLP sepsis models^31,32^. For example, NETs formed from activated neutrophils during sepsis can trap bacteria and fungi to contain the infection, while also inducing local and distant inflammation through DAMPs like cfDNA^10,33,34^. PAD4, a crucial enzyme, regulates NET formation through histone citrullination^14^. Consequently, neutrophils lacking PAD4 fail to produce NETs, even when stimulated by chemokines, LPS, or bacteria^14,35^.

**Fig. 8:**
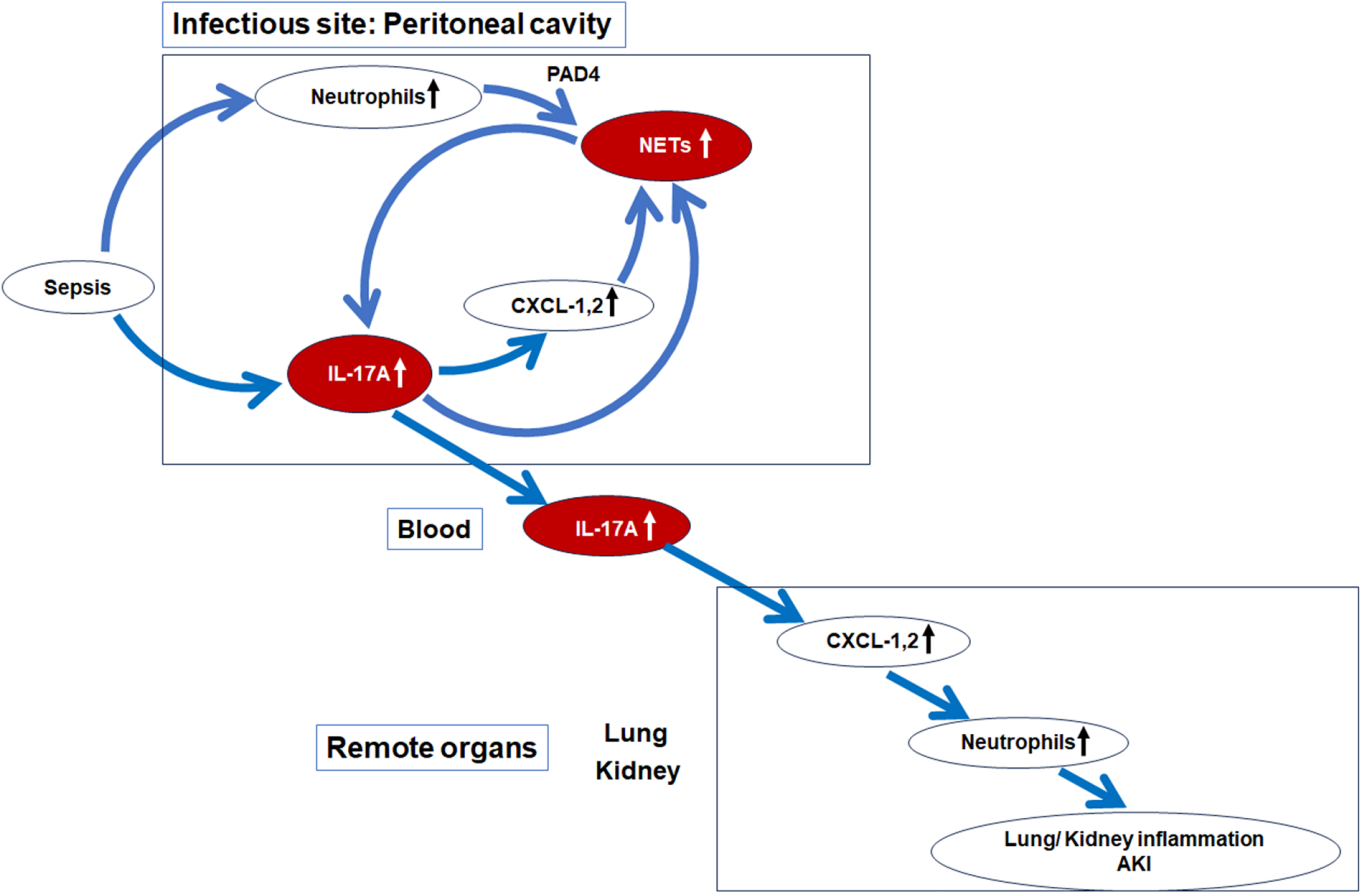
Schematic presentation of the pathway from NET formation and IL-17A production in the peritoneal cavity to remote organ injuries in the CLP model. CLP upregulated neutrophil accumulation and PAD4 mediated NET formation in the peritoneal cavity (Fig. 4). CLP increased IL-17A production in PLF and plasma, which was significantly decreased by *Pad4* knockout (Fig. 2 C and D). Knockout of *Il-17a* significantly decreased CXCL-1 and -2 production in PLF after CLP (Fig. 5A). Knockout of *Il-17a* decreased NET formation in peritoneal cavity after CLP (Figs. 5C and 6A–D). Recombinant IL-17A, CXCL-1 and -2 upregulated NET formation in PLF cells collected from WT mice at 3 h after CLP ex vivo (Fig. 6E and F). *Pad4*KO as well as *Il-17a*KO significantly decreased CXCL-1 and -2 production in kidney and lung, neutrophil infiltration into kidney and lung, and AKI after CLP (Figs. 1 and 3). *Pad4*KO significantly improved survival after CLP (Fig. 1A). Adoptive transfer of WT neutrophil into *Pad4*KO mice restored IL-17A production in PLF and plasma, CXCL-1 and -2 production in kidney and lung, neutrophil infiltration into kidney and lung, and AKI (Fig. 7).

In this study, we explored the function of ‘local’ peritoneal NETs formed at site of infection, and their impact on distant organ function. The main findings of this paper are: 1) elimination of NETs by knockout of *Pad4* improved survival and AKI after CLP; 2) NETs were detected only in the peritoneal cavity, not in plasma or distant organs; 3) knockout of *Pad4* suppressed neutrophil infiltration into remote organs via a peritoneal IL-17A and distant organ CXCL-1/CXCL-2 pathway; 4) knockout of *Il-17a* suppressed NET formation and CXCL-1/CXCL-2 production in peritoneal cavity after CLP and recombinant IL-17A, CXCL-1, or CXCL-2 promoted NET formation in PLF cells; and 5) adoptive transfer of peritoneal WT neutrophils restored the peritoneal IL-17A and distant organ CXCL-1/CXCL-2 pathway in *Pad4*KO mice, leading to neutrophil infiltration into remote organs and remote organ injury. Our findings are summarized in Fig. 8.

*Elimination of NETs by Knockout of Pad4 improves survival and AKI after CLP.* We found that *Pad4* knockout improved survival and AKI in a CLP model in which the cecum was punctured with 21-gauge needles, and animals were treated with fluids (1 ml of 2/3 normal saline) and antibiotics (s.c.) every 12 h for 7 days. Survival on day 4 after CLP was 53% for WT mice vs. 85% for *Pad4*KO and on day 7 was 47% for WT mice vs. 80% for *Pad4*KO. NETs defend against bacteria by trapping or killing them^11^. Inhibiting NETs during severe infections could be detrimental for sepsis. Interestingly, *Pad4* knockout did not change the peritoneal bacterial count 18 h after CLP. Similar positive results on CLP survival have been seen with Cl-Amidine, a non-specific PAD inhibitor or antibodies against H3Cit^22,23,36^. Our findings contrast with Martinod et al.^19^, who found no effect of *Pad4* knockout in a mild CLP model (using 21-gauge needles, no antibiotics, 75% survival at 4 days, with 7–8 animals per group), and worse outcomes in a severe CLP model (18-gauge needles, with antibiotics, no survival at 10 days). They only partially replaced surgical fluid losses (0.5ml of normal saline once immediately after surgery)^19^. Our model resembles their mild CLP model but includes antibiotics, more fluid, and longer intermittent fluid resuscitation. Variations in CLP severity, fluid resuscitation, antibiotic administration (none, subcutaneous, or intraperitoneal), and our larger sample size (19 to 20 mice per group) could explain the differing results between laboratories. Interestingly, even in their severe CLP model, knockout of *Pad4* did not increase bacterial loads in the blood, liver, or lungs at 24 h post-CLP^19^. However, NETs might have aided in reducing bacterial abundance later^19^. Losing NET protection against bacteria could be harmful in severe CLP models, yet dampen excessive inflammation in milder CLP models.

*NET formation was detected only in the peritoneal cavity, and not in plasma or distant organs.* Due to the positive effect of PAD4 inhibition/deletion on reducing distant organ damage and improving survival, we anticipated widespread NET presence, contributing to multiple organ failure. The detrimental effects of NETs in sepsis are attributed to tissue-based NET formation, intravascular coagulation promotion, and the release of enzymes like neutrophil elastase and serine proteases during NETosis^10^. Initially, we hypothesized that intraperitoneal NETs would induce distant NET formation in remote organs via the production of proinflammatory cytokines, chemokines, and damage-associated molecular patterns (DAMPs) like nuclear and/or mitochondrial cfDNA. Surprisingly, we discovered that NETs were confined to the peritoneal cavity and not detected in plasma or other distant organs (lung, liver, kidney, spleen). The location of NETs following sepsis is complex, and it appears to be quite sensitive to the severity of sepsis. NETs have been identified in lungs, kidneys, and plasma after CLP; however, these CLP models were considerably more severe. For example, H3Cit-positive cells were detected in the lungs post-CLP^37–41^—but survival was 0% at 24 h^40^ or 15% at 48 h^41^, compared to 100% at 24 and 48 h in our model. H3Cit-positive cells have been found in the kidney at 24 or 48 h after CLP^42^; however, the model was more severe than our model, with 40% survival in WT mice at 24 h post-CLP^42^. Elevated serum H3Cit levels have also been detected post-CLP^22,43^; again, in more severe sepsis models. Yuzi et al. reported survival rates after CLP: around 60% at 24 h and 0% at 60 h post-CLP^22^. In contrast, Bethany et al. found that plasma H3Cit levels were not increased by CLP surgery compared to sham, consistent with our results^23^. Their CLP model showed 80% survival at 24 h and 50% at 60 h, indicating a milder outcome compared to Yuzi’s model, which exhibited elevated blood H3Cit after CLP, and relatively similar to ours^22,23^. These findings imply that NETs may form in lungs, kidneys, or blood during severe CLP. However, in a less severe sepsis model, we found that NET formation in PLF induces (see below) lung inflammation or kidney injury even without NET formation in blood or these remote organs.

*Knockout of Pad4 suppressed neutrophil infiltration into remote organs *via* a peritoneal IL-17A and distant organ CXCL-1/CXCL-2 pathway.* We then investigated the mediator(s) from NETs circulating systemically causing organ injury. *Pad4* knockout reduced IL-17A in PLF and plasma after CLP. Interestingly, a myeloid-specific *Pad4* deletion greatly reduced IL-17A production and IL-17A-producing αβ and γδ T cells from the aorta of an atherosclerosis model^29^. NET-derived histones stimulated TLR2 on T cells, inducing STAT3 phosphorylation, which promoted differentiation into Th17 cells and enhanced IL-17A production^28^. Although γδ T cells—not αβ T cells (including Th17 T cells)—have been reported as the primary source of systemic IL-17A production in sepsis^44^, NETs could promote IL-17A production by γδ T cells in sepsis via a similar molecular mechanism. Furthermore, *Pad4* and *Il-17a* knockout notably reduced CXCL-1 and CXCL-2 levels in lung and kidney and decreased neutrophil infiltration. This indicates IL-17A, promoted by NET formation, is vital for CXCL-1, CXCL-2 production, and neutrophil infiltration in distant organs post-CLP. IL-17A stimulates epithelial cells to produce chemokines, and promotes myeloid cell mobilization to inflammatory sites^30^. Notably, CXCL-8 in humans and its functional homologs, CXCL-1 and CXCL-2 in mice, are potent inducers of neutrophil migration into inflamed tissues^45^. *Il-17a* knockout reduces CXCL-1 and -2 levels, neutrophil infiltration, and kidney impairment after CLP^46^. Thus, IL-17A plays a crucial role in distant organ CXCL-1/CXCL-2 production and neutrophil infiltration.

*Knockout of Il-17a suppressed NET formation and CXCL-1/CXCL -2 production in peritoneal cavity after CLP and recombinant IL-17A, CXCL-1, or CXCL-2 promoted NET formation in PLF cells.* After finding NET formation promoted the IL-17A pathway in the CLP model, we assessed if the IL-17A pathway affects NET formation. *Il-17a* knockout decreased H3Cit, CXCL-1, and CXCL-2 levels in PLF after CLP in vivo and reduced NET formation in PLF cells after CLP ex vivo. Additionally, recombinant IL-17A, CXCL-1, or CXCL-2 promoted NET formation in PLF cells ex vivo. Previous studies support our results, indicating IL-17A, CXCL-1, or CXCL-2 can promote NET formation^40,47–49^. *Pad4* knockout markedly reduced IL-17A production in PLF or plasma, while *Il-17a*KO partially decreased NET formation in PLF, though it was statistically significant. In our CLP model, factors beyond IL-17A and the CXCL-1/CXCL-2 pathway in the peritoneal cavity may also influence intraperitoneal NET formation. However, these findings suggest a vicious cycle of IL-17A, CXCL-1/CXCL-2 pathway, and NETs in the peritoneal cavity, potentially exacerbating CLP-induced organ inflammation/injury.

*Adoptive transfer of peritoneal WT neutrophils restored upregulated the peritoneal IL-17A and distant organ CXCL-1/CXCL-2 pathway in Pad4KO mice, leading to neutrophil infiltration into remote organs and remote organ injury.* In adoptive transfer experiments, we examined the role of intraperitoneal NET formation in distant organ injury/inflammation in CLP. Transferring WT peritoneal neutrophils into *Pad4*KO mice reversed the attenuation seen with *Pad4* knockout, unlike *Pad4*KO neutrophils. This reversal also included IL-17A levels in PLF and plasma, CXCL-1 and CXCL-2 levels in kidney and lung, neutrophil infiltration into kidney and lung, and AKI. These findings support our hypothesis that intraperitoneal NET formation critically contributes to remote organ inflammation/injury via a peritoneal IL-17A and distant organ CXCL-1/CXCL-2 pathway in our mouse CLP model.

## Limitations

Several limitations exist in this study. First, we utilized mice with systemic *Pad4* knockout. PAD4 is expressed not only in neutrophils but also in other immune cells like eosinophils, monocytes, macrophages, and natural killer cells^50^. Furthermore, extracellular traps are known to occur not only on neutrophils but also on mast cells, eosinophils, basophils, and monocytes/macrophages^51,52^. Although the large majority of immune cells in the peritoneal cavity after CLP were neutrophils, the extracellular traps in our experiments may have included extracellular traps derived from these immune cells other than neutrophils, which could be validated by staining for neutrophil markers. Given the use of whole-body *Pad4*KO mice, we cannot ascertain if the effect stems from neutrophil PAD4. To address this, we conducted adoptive transfer of purified neutrophils from WT to *Pad4*KO mice, indicating the significance of intraperitoneal neutrophil PAD4 in the inflammation/injury pathogenesis in remote organs. Secondly, detecting neutrophils with naphthol AS-D chloroacetate esterase, and identifying NETs only by citH3 staining alone or by measuring citH3 in tissue homogenates with ELISA kits—may result in missing important mechanistic information and could potentially underestimate NET formation in distant organs, i.e. kidneys and lungs. Flow cytometry of immune cells from these organs could provide more precise insight into this process. Thirdly, PAD4 has other effects beyond its critical role in NET formation. It mediates apoptosis, inflammation, and pluripotency^50^. PAD4 promoted thrombin activity via antithrombin inactivation in rheumatoid arthritis^53^. The function of PAD4 other than NET formation might contribute to the beneficial effect of knockout of *Pad4* on our CLP model. Fourth, complete gender equalization of transplanted neutrophils could not be achieved. Neutrophil function is known to be influenced by sex differences. Multiple studies have reported that female mouse neutrophils demonstrate higher bactericidal activity, activation markers (e.g., CD11b, CR3), and cytokine responses in males^54–56^. Estrogen has been reported to promote NET formation^57^, while another study suggested it might also suppress neutrophil migration and activation (e.g., superoxide production)^58^. Although no apparent differences were observed between male and female neutrophils regarding AKI or IL-17A production following neutrophil adoptive transfer in our model, these factors could have influenced the result. Fifth, in adoptive transfer experiments, we could not rule out the possibility that neutrophils migrated directly to distant organs. Although the data suggest that NETs derived from WT neutrophils mobilize the recipient mouse’s neutrophils to distant organs via the IL-17/CXCL1 axis, the possibility remains that the donor’s WT neutrophils themselves migrated from the peritoneum to distant organs and contributed to the damage there. Another limitation is interspecies differences in NET formation. Human neutrophils form NETs more readily than mouse neutrophils^10,59,60^. This implies that NETs might more likely form in peripheral blood or distant organs in humans than in mice. Further research is necessary to understand the role of local NET formation to distant organ injury via local IL-17A and remote CXCL-1/ CXCL-2 in humans.

In summary, this study is the first to elucidate how intraperitoneal NET formation can trigger distant organ injury and inflammation via the IL-17A and CXCL-1/CXCL-2 pathway without remote organ NET formation after CLP. *Pad4* knockout improved survival in a clinically relevant abdominal sepsis model with broad-spectrum antibiotics and fluids resuscitation. *Pad4* knockout reduced IL-17A production in PLF and plasma. Both *Pad4* and *Il-17a* knockout ameliorated AKI and reduced neutrophil infiltration into the kidney and lung by lowering CXCL-1/CXCL-2 levels, known downstream factors of IL-17A, in these organs. Adoptive transfer of WT neutrophils restored CLP-induced AKI and neutrophil infiltration into kidney and lung, as well as CXCL-1 and CXCL-2 levels in these organs, and IL-17A levels in PLF and plasma attenuated by *Pad4* knockout. These findings suggest a pathway from peritoneal NET formation to distant organ injury/inflammation via peritoneal IL-17A production and distant organ CXCL-1/CXCL-2. While NETs promoted IL-17A production in PLF and plasma, we demonstrated reciprocally that IL-17A or CXCL-1 and CXCL-2 promoted NET formation in PLF after CLP. These results highlight a potential vicious cycle among NET formation, IL-17A, and CXCL-1/CXCL-2 amplifying organ injury and inflammation in sepsis. Disrupting this cycle by inhibiting NET formation or IL-17A could be promising therapeutic strategies for sepsis treatment in carefully selected patients.

## Methods

### Animals

All animal studies were approved by the NIDDK Animal Care and Use Committee (K100-KDB). All experiments were performed in accordance with relevant guidelines and regulations and with ARRIVE guidelines. *Il-17a*KO (Strain #:016,879), *Pad4*KO mice (Strain #:030,315), and C57BL/6J WT controls (Strain #:000,664) were obtained from Jackson Laboratory (Bar Harbor, ME). Mice had an acclimation period of at least 7 days prior to use for any experiments.

### CLP

CLP was performed as previously described [^25^]. Briefly, 9–12-week-old male mice were anesthetized (isoflurane 5% for induction and 3% to maintain anesthesia). The cecum was ligated at 1 cm from the cecal tip, punctured twice with a 21-gauge needle, and gently squeezed to express a 1-mm column of cecal material. Sham surgeries were identical (without cecal ligation and puncture). Post-surgery, mice received subcutaneous Buprenorphine ER (1.2 mg/kg) and intraperitoneal normal saline (1.0 mL). Mice were euthanized 3 or 18 h post-CLP. Blood and tissues were collected. For ELISA analysis, 2 mL of PBS containing 2 mM EDTA was injected intraperitoneally, followed by collection of PLF. For collection of PLF cells for flow cytometry or immunocytochemistry, 4 mL of PBS containing 2 mM EDTA was injected intraperitoneally, followed by collection of PLF.

### Adoptive transfer of isolated neutrophils into CLP treated mice

Male/female WT or female *Pad4*KO mice (9–12 weeks old) underwent CLP surgery. PLF was collected at 18 h post-CLP. Neutrophils from PLF were purified using a mouse Neutrophil Isolation Kit (Miltenyi Biotec GmbH, Bergisch-Gladbach, Germany), labeled with anti-mouse Ly6G Pacific Blue and anti-mouse CD11b APC/Cy7 antibodies, and analyzed via flow cytometry (~ 95% pure Ly6G + CD11b + cells; Supplemental Fig. 4B). Neutrophils were counted using Countess II (Invitrogen, Carlsbad, CA). Neutrophils (10^6^) were intraperitoneally injected into male *Pad4*KO mice immediately after CLP. Mice were euthanized 18 h post-CLP, and tissue specimens, blood, and PLF were harvested. Initially, we planned to use only male mice as donors. However, the large number of male *Pad4*KO mice required (all recipients and half of the donors needed to be *Pad4*KO) meant that we could not prepare a sufficient number of male *Pad4*KO mice. Therefore, we had no choice but to use female *Pad4*KO mice as donors. To match the *Pad4*KO donors, we attempted to prepare female WT mice as donors. However, ultimately, 3 of the 12 recipients transplanted with WT neutrophils received male neutrophils, while all 7 recipients transplanted with *Pad4*KO neutrophils received female neutrophils. Although there remain concerns regarding differences between transplanted male and female neutrophils, no significant differences in AKI or IL-17A production levels were observed between *Pad4*KO mice transplanted with WT male neutrophils and those transplanted with WT female neutrophils in this experiment (Supplemental Fig. 10).

### Survival study

Mice were monitored every 6–12 h post-CLP, with euthanasia of survivors at 168 h. Antibiotic and fluid resuscitation began 6 h post-CLP via subcutaneous injection of imipenem/cilastatin (14 mg/kg) in 1 mL of 2/3 NS, repeated with 7 mg/kg in 1 mL of 2/3 NS every 12 h. Additional doses of Buprenorphine ER were given at 72 and 144 h post-CLP.

### Collection of PLF cells

PLF cells were harvested after injection of 4 mL PBS with 2 mM of EDTA into the peritoneum and centrifuged for 5 min at 500 × g 4 ℃. The cells were resuspended in MACs buffer (Miltenyi Biotech, Auburn, CA) for flow cytometry, or RPMI-1640 medium (containing 10 mM HEPES and 0.5% BSA) without phenol red for immunocytochemistry or NET visualization using SYTOX Green (Invitrogen).

### In vitro NET generation

PLF cells were collected and pooled from 4 WT mice 3 h after CLP. 5 × 10^4^ cells were transferred to a poly-L-lysine-coated coverslip (Corning) in 24 well plates, stimulated with 20 ng/ml mouse recombinant IL-17A, CXCl-1, -2 protein (R&D Systems), or 100 nM of phorbol myristate acetate (PMA) for 3 h in a humidified incubator (37 °C, 5% CO_2_)^61^. Initially, we applied stimuli to PLF cells collected 18 h after CLP, but it was difficult to detect any stimulation, likely because the cells were already highly activated. Therefore, we collected PLF cells 3 h after CLP, because they were less activated. The coverslips were stained with anti-Histone H3 (citrulline R2 + R8 + R17) antibody (Abcam) and Hoechst 33,342 (Thermo Fisher Scientific), see also Supplemental Methods. 30 images were obtained using a confocal microscope (Zeiss LSM780, Zeiss) from different areas from 2–3 slides in each group. These images were analyzed for % NETs formed and NET extension.

### Statistical analyses

The results are expressed as means ± standard error of means (SEM). The normality of the data distribution was visually checked using histograms. Differences between two groups were analyzed using unpaired Welch’s t-tests and differences among three or more groups were analyzed using one-way ANOVA followed by Tukey’s multiple comparisons test or two- way ANOVA followed by Šídák test. Mouse survival was depicted with Kaplan–Meier curves (log-rank test). All statistical calculations were performed using GraphPad Prism software (GraphPad Software Inc., La Jolla, CA). *P* values ≤ 0.05 were considered a statistically significant.

Further details are provided in Supplemental Methods.

## Supplementary Information

Below is the link to the electronic supplementary material.

Supplementary Material 1

Supplementary Material 2

Supplementary Material 3

Supplementary Material 4

Supplementary Material 5

Supplementary Material 6

Supplementary Material 7

Supplementary Material 8

Supplementary Material 9

Supplementary Material 10

Supplementary Material 11

Supplementary Material 12

Supplementary Material 13

## Data Availability

Underlying data are published on Mendeley Data (Reserved https://doi.org/10.17632/y2tbj7h96t.1).
