# Supplementary material for: Peritoneal neutrophil extracellular traps contribute to septic AKI via peritoneal IL-17A and distant organ CXCL-1/ CXCL-2 pathway in abdominal sepsis
Source: Sci Rep. 2026 Jan 27;16:5446. doi: 10.1038/s41598-025-34770-1 (PMC12886817; doi:10.1038/s41598-025-34770-1)
Supplement: Supplementary file 1 — Supplementary Material 1 [file 41598_2025_34770_MOESM1_ESM.pdf]

Supplemental methods

Bacterial counts

PLF was collected post i.p. injection of 2 ml sterile PBS 18 h post-CLP surgery. Serial dilutions were plated on 5% sheep blood agar (BD, Franklin Lakes, NJ), incubated at 37°C for 24 h, and colonies were counted.

Functional and pathological assessment of the kidney

BUN levels were measured using QuantiChrom™ Urea Assay Kit (DIUR-100) (BioAssay Systems, Hayward, CA). Tubular damage was assessed using periodic acid-Schiff stained sections[1]. Briefly, cortical tubular vacuolar degeneration was measured at 400× magnification, with 50 randomly selected tubules assessed per mouse and averaged. The degree of kidney damage was graded as follows: 0, normal; 1, < 25% damage; 2, 25%–50% damage; 3, 50%–75% damage; 4, 75%–100% damage.

Immunohistochemistry for Citrullinated histone H3

Tissues were fixed in 10% formalin and paraffin embedded. Sections (5 μm thick) were cut, deparaffinized, and incubated in sodium citrate for antigen retrieval. After nonspecific binding sites were blocked with goat serum, sections were incubated with rabbit anti-Histone H3 (citrulline R2 + R8 + R17) antibody (#ab5103, Abcam, Cambridge, UK) overnight at 4°C.

Subsequently, sections were incubated with horseradish peroxidase–conjugated goat anti-rabbit antibody (Agilent Dako, Santa Clara, CA). Immunostaining was performed using 3,3′-diaminobenzidine tetrahydrochloride (Sigma-Aldrich, St. Louis).

Chloroacetate esterase stain to detect neutrophil in tissue sections

Tissues were fixed in 10% formalin and paraffin-embedded. Sections (5 μm) were deparaffinized and stained with a naphthol AS-D chloroacetate esterase kit (Millipore Sigma, Burlington, MA) following the manufacturer's instructions to identify neutrophils. Neutrophils were counted in 10 fields at 400x magnification and averaged per mouse.

Immunocytochemistry

PLF cells were obtained from WT, *Pad4*KO, or *Il-17a*KO mice at 18 h post-CLP. 50 μl cell suspensions (5×10^4 cells) were added to a poly-L-lysine-coated coverslip (#354085, Corning, Corning, NY) in each well of a 24-well plate, then incubated for 3 h in a humidified incubator (37°C, 5% CO2) without *in vitro* stimulation. The cells were fixed with 4% paraformaldehyde. After nonspecific binding sites were blocked with PBS containing 1% goat serum and 1% BSA, coverslips were incubated with rabbit anti-Histone H3 (citrulline R2 + R8 + R17) antibody (#ab5103, Abcam, Cambridge, UK) overnight at 4°C, and then incubated with Goat anti-Rabbit IgG (H+L) Cross-Adsorbed Secondary Antibody, Alexa

Fluor™ 488 (#A-11008/ Lot 982425, Invitrogen, Carlsbad, CA) for 30 min at 37°C. After PBS washing, cells were stained with 10 µg/ml Hoechst 33342 (Thermo Fisher Scientific, Waltham, MA) for 15 min at room temperature. The coverslips were then imaged using a confocal microscope (Zeiss LSM780, Zeiss, Oberkochen, Germany). Five images from different areas of each coverslip were captured using a 40× objective, and analysis was performed using Fiji/ImageJ software (National Institutes of Health, Bethesda, MD). Citrullinated histone H3-positive structures extruded from cells were considered NET events. NET events were divided by total cell number and averaged per mouse (% NETs). The degree of NET extension was also calculated by normalizing the citrullinated histone H3-positive area by the number of cells and averaged per mouse.

NET visualization using SYTOX Green

A Cytation 5 Cell Imaging Multi-Mode Reader (BioTek-U.S., Winooski, VT, USA) was used to assess NET extension in PLF cells. 5 X 10^5 PLF cells per well were seeded on 24-well plates coated with 1% BSA and incubated at 37°C for 2 h. 200 nM SYTOX Green (Invitrogen) and 4 µg/ml Hoechst 33342 (Thermo Fisher Scientific) were added to the plates 15 min before analysis. Eight 1973 X 1457 µm images per well for designated areas were automatically collected, processed, and analyzed for NET extension (SYTOX Green- positive area normalized by cell count) by Cytation 5 using Gen 5 software (Bio Tek-U.S.).

Kidney tissue dissociation for flow cytometry analysis

Kidneys were harvested from mice after left ventricular perfusion with 20 ml of ice-cold PBS and minced with a razor blade on ice. Minced tissues were incubated in HEPES-buffered RPMI-1640 containing 0.2125 mg/mL of Liberase (Roche, Switzerland) and 100 U/ml of DNase I (Roche) at 37 °C for 25 min. Cell suspensions were filtered through 40 μm nylon mesh (BD Falcon, Bedford, MA), centrifuged at 500 x g for 5 min at 4 °C, and resuspended in MACS buffer (Miltenyi Biotech).

Flow cytometry analysis

PLF cells or kidney tissues were stained with anti-mouse CD45, Ly6G, CD3, CD4, CD8, CD11b, CD19, CD64, γδTCR, NK1.1, and/or F4/80 antibodies as described in detail in Table S1. Mouse BD Fc Block™ (1:25, #553142, BD Biosciences, San Diego, CA) was used to block the Fc-mediated binding of antibodies. Cell viability was evaluated with BD Pharmingen™ 7-AAD (BD Biosciences) or Zombie Violet™ Fixable Viability Kit (1:300, #423113, BioLegend, San Diego, CA) according to the manufacturer's instructions. Absolute cell counts in samples were counted by using CountBright Absolute Counting beads (#C36950, Invitrogen) and standardized by kidney weight or total PLF volume. Sample

acquisition was performed on a BD FACSymphony™ (BD Biosciences) or BD LSRFortessa™ Cell Analyzer (BD Biosciences). The data was analyzed by FlowJo software (Tree Star, Ashland, OR).

Cytokines

IL-17A, CXCL-1, and CXCL-2 concentration in plasma, PLF, and tissue homogenates (normalized by total [protein]) were measured using the corresponding mouse enzyme-linked immunosorbent assays (ELISA) kits (R&D Systems, Minneapolis, MN) according to the manufacturer's instructions.

ELISA for Citrullinated Histone H3

Kidneys were sliced and placed in microtubes containing RIPA (radioimmunoprecipitation assay) buffer, which included protein inhibitors and phosphatase inhibitors. They were then homogenized using a Biomasher (Funakoshi, Tokyo, Japan). Lungs and spleens were homogenized with Hard Tissue Homogenizing Precellys® CK28 Lysing Kit (Bertin Copr., Rockville, MD) in RIPA buffer containing protein inhibitor and phosphatase inhibitor at 6500 rpm for 30 s, repeated three times with 15-s intervals between each run. After incubating these homogenates (kidney, lung, spleen) on ice for 30 min, they were centrifuged at 13,000 rpm at 4°C for 15 min. The supernatant was collected and subjected to ELISA analysis. Citrullinated

Histone H3 levels in plasma, PLF, and tissue homogenates (normalized by total [protein]) were measured using Citrullinated Histone H3 (Clone 11D3) ELISA Kit (#501620, Cayman Chemical, Ann Arbor, MI) according to the manufacturer's instructions.

Blinding and Randomization

A scientist was blinded by another researcher to the strains or treatment groups while CLP surgery and subsequent analysis was in progress (including BUN and histology). The order of mice to undergo surgery was randomly determined using the standard = RAND() function in Microsoft Excel.

Sample size

Sample size was determined prior to conducting the study. The experimental unit was a single animal. It was decided in advance to exclude mice that suffered unintended bleeding or organ damage during the CLP procedure, and all other mice were included in the study. The sample size for the survival study was n=20 for each group. One mouse with a cecal injury during the CLP procedure was excluded from the survival study. For CLP studies other than survival studies, the CLP group n=9-12 and sham group n=5. In the adoptive transfer experiment, the sample size was initially determined as neutrophil transfer group n=9-12 and vehicle group

n=5. However, the sample size for the Pad4KO mice injected with Pad4KO neutrophils was n=7 because the number of Pad4KO neutrophils obtained by neutrophil isolation was lower than expected (originally determined as n=9).

1. Naito Y, Tsuji T, Nagata S, *et al.* IL-17A activated by Toll-like receptor 9 contributes to the development of septic acute kidney injury. *American Journal of Physiology - Renal Physiology* 2020; **318:** F238-F247.

Supplemental Figure 1. Representative images of periodic acid-Schiff (PAS) stained renal tissue of WT or *Pad4*KO mice at18 h after sham or CLP surgery. Original magnification, ×400. Scale bars = 20 μm.

Supplemental Figure 2. (A and B) Representative images of neutrophils (arrows) in kidney (A) and lung (B) of WT or *Pad4*KO mice at 18 h after sham or CLP surgery using naphthol AS-D chloroacetate esterase staining. Neutrophils are stained pink. Original magnification, ×400. Scale bars = 20 μm.

Supplemental Figure 3. Knockout of *Pad4* or *Il-17a* did not alter bacterial count in PLF at 18 h after CLP. (A, B) Bacterial count in PLF from WT (n=5) or *Pad4*KO (n=5) (A)/ WT (n=5) or *Il-17a*KO (n=5) (B) at 18 h after CLP. Values represent the means ± SEM. ns: not significant.

Supplemental Figure 4. Representative images of PAS-stained renal tissue of WT or *Il-17a*KO mice at 18 h after sham or CLP surgery. Original magnification, ×400. Scale bars = 20 μm.

Supplemental Figure 5. (A and B) Representative images of neutrophils (arrows) in kidney (A) and lung (B) of WT or *Il-17a*KO mice at 18 h after sham or CLP surgery using naphthol AS-D chloroacetate esterase staining. Neutrophils are stained pink. Original magnification,

×400. Scale bars = 20 μm.

Supplemental Figure 6. Flow cytometry analysis revealed that CLP surgery upregulated neutrophil infiltration into kidney through the IL-17A pathway. (A) Representative images of flow cytometry analysis for CD11b+ Ly6G+ neutrophils in kidney at 18h after sham or CLP surgery. Living single CD45+ cells (CD45 positive, 7-AAD negative) were gated. Then CD11b and Ly6G expression was analyzed in this population. (B) Number of CD11b+ Ly6G+ neutrophil in kidney at 3 and 18 h after sham or CLP surgery (n=6 per group). (C) Representative images of flow cytometry analysis for CD11b+ Ly6G+ neutrophils in kidney of WT or *Il-17a*KO mice at 18h after CLP surgery. (D) Number of CD11b+ Ly6G+ neutrophils in kidney of WT or *Il-17a*KO mice at 3 and 18 h after CLP surgery (n=6 per group). Values represent the means ± SEM. *P < 0.05, **P < 0.01, *** P < 0.001, **** P < 0.0001.

Supplemental Figure 7. Knockout of *Il-17a* did not significantly alter the absolute number or percentage of neutrophil infiltration into peritoneal cavity at 18 h after CLP. (A) Total number of Ly6G+ neutrophils in PLF from WT or *Il-17a*KO mice at 18h after CLP surgery. Living (Zombie Violet negative), single CD45+ cells were gated. Then Ly6G expression was analyzed in this population (n=9 per group). (B) Percentage of Ly6G+ neutrophils in living CD45+ cells in PLF from WT or Il-17aKO mice at 18h after CLP. Values represent the means ± SEM. ns:

not significant.

Supplemental Figure 8. Adoptive transfer experiments of WT neutrophils into *Pad4*KO mice after CLP. (A) Experimental strategy. Neutrophils were isolated from the peritoneal cavity of WT or *Pad4*KO mice at 18h after CLP surgery. WT (n = 12) or *Pad4*KO (n = 7) neutrophils, or vehicle (n = 5) were adoptively transferred to *Pad4*KO mice intraperitoneally, immediately after CLP surgery. (B) The percentage of neutrophils among living PLF cells--before and after neutrophil isolation--for neutrophil adoptive transfer. Living (7AAD negative) single cells were gated. Then CD11b and Ly6G expression was analyzed in this population. Numbers in the lower right show the percentage of CD11b+ Ly6G+ neutrophils in a single living PLF cells.

Supplemental Figure 9. (A) Representative images of periodic acid-Schiff (PAS) stained renal tissue of *Pad4*KO mice at 18 h after CLP surgery injected with WT (n = 12) or *Pad4*KO (n = 7) neutrophils, or vehicle (n = 5). Original magnification, ×400. (B) Representative images of neutrophils (arrows) in kidney of *Pad4*KO mice at18 h after CLP surgery injected with WT (n = 12) or *Pad4*KO (n = 7) neutrophils, or vehicle (n = 5) using naphthol AS-D chloroacetate esterase staining. Neutrophils are stained pink. Original magnification, ×400. (C) Representative images of neutrophils (arrows) in lung of *Pad4*KO mice at 18 h after CLP surgery injected with WT (n = 5) or *Pad4*KO (n = 5) neutrophils using naphthol AS-D

chloroacetate esterase staining. Neutrophils are stained pink. Original magnification, ×400. (D) Neutrophils were counted in 10×400 fields/mouse and averaged. Values represent the means ± SEM. *P < 0.05. Scale bars = 20 μm.

Supplemental Figure 10. Differences between transplanted WT male, WT female, and *Pad4*KO female neutrophils, in AKI or IL-17A production levels in PLF and plasma in adoptive transfer experiments. Plasma BUN levels (left), or IL-17A concentration in PLF (middle), and plasma (right) in *Pad4*KO mice at 18 h after CLP surgery injected with WT male (n = 3), WT female (n = 9), or *Pad4*KO female (n = 7) neutrophils. Values represent the means ± SEM. *P < 0.05. ns: not significant.
