# Supplementary figures and images for: Peritoneal neutrophil extracellular traps contribute to septic AKI via peritoneal IL-17A and distant organ CXCL-1/ CXCL-2 pathway in abdominal sepsis
Source: Sci Rep. 2026 Jan 27;16:5446. doi: 10.1038/s41598-025-34770-1 (PMC12886817; doi:10.1038/s41598-025-34770-1)

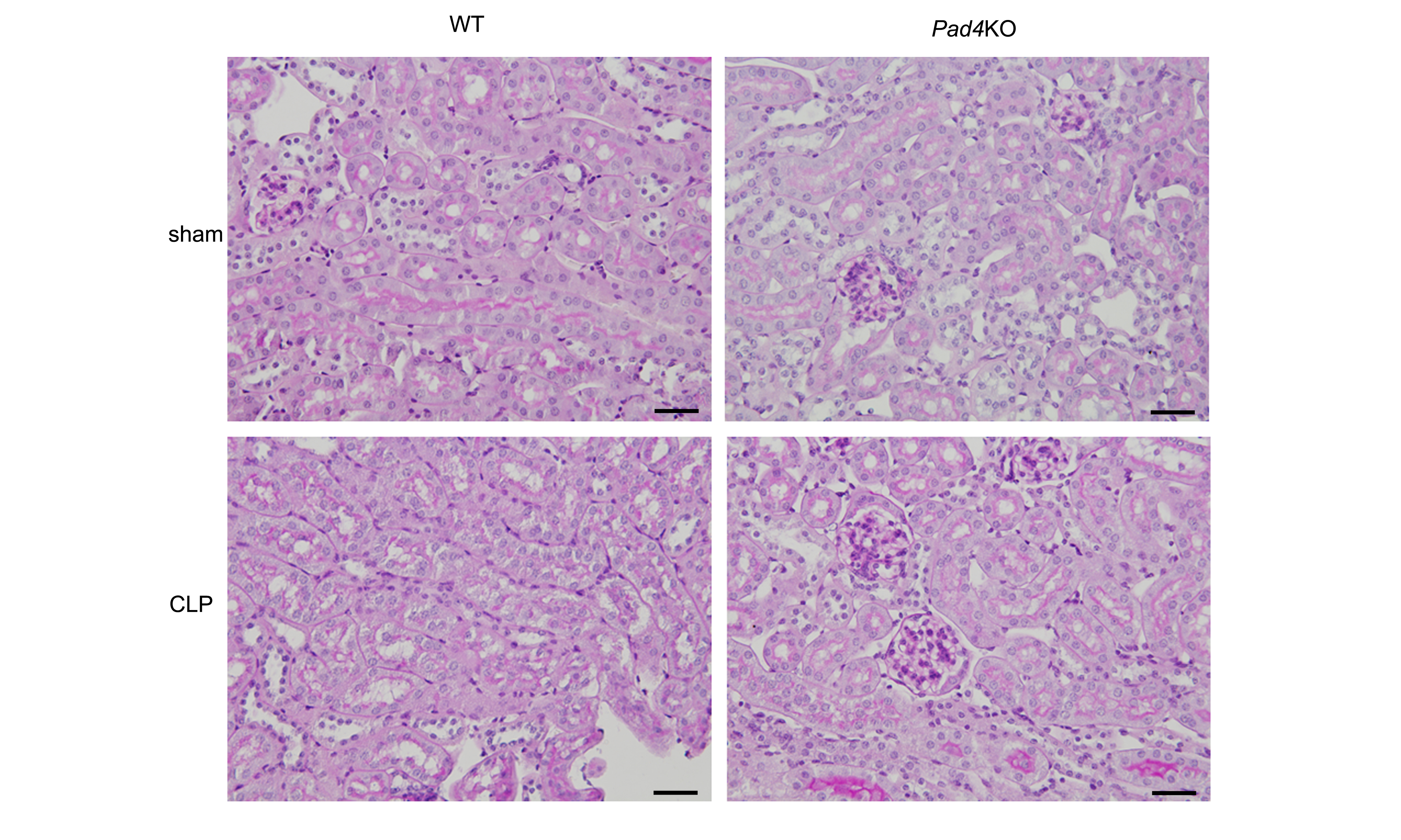

Supplement: Supplementary file 2 — Supplementary Material 2 [file 41598_2025_34770_MOESM2_ESM.jpg]

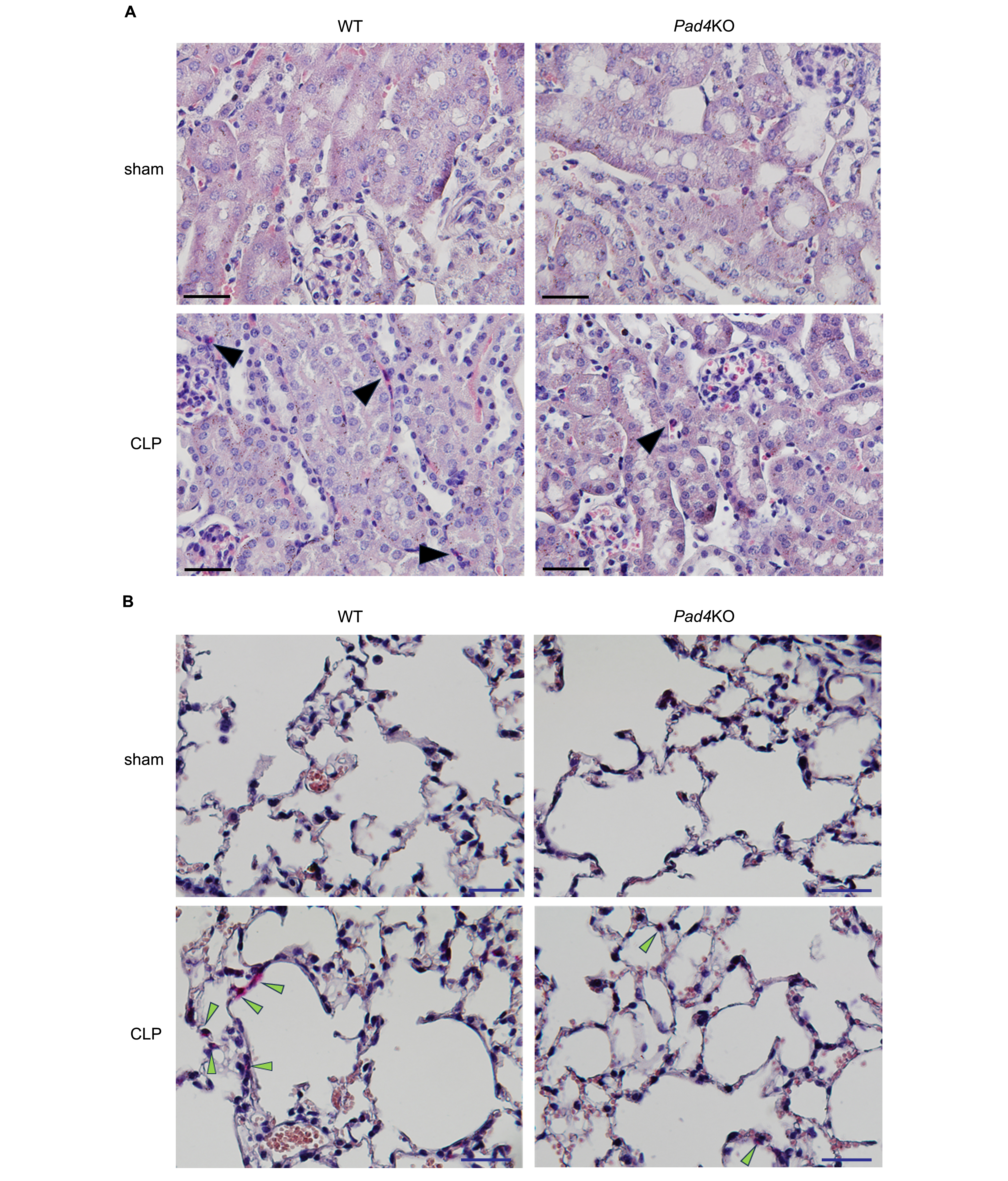

Supplement: Supplementary file 3 — Supplementary Material 3 [file 41598_2025_34770_MOESM3_ESM.jpg]

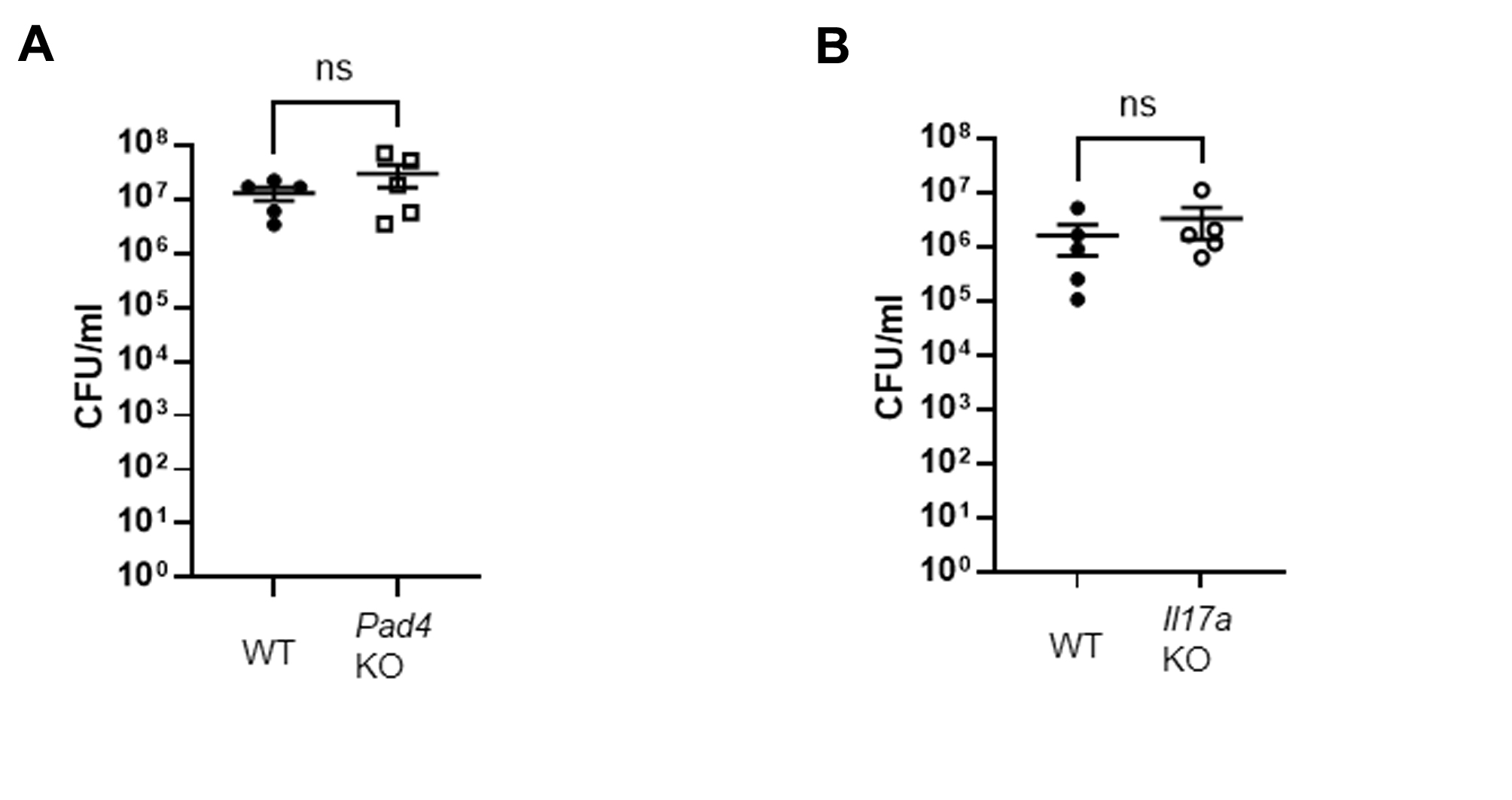

Supplement: Supplementary file 4 — Supplementary Material 4 [file 41598_2025_34770_MOESM4_ESM.jpg]

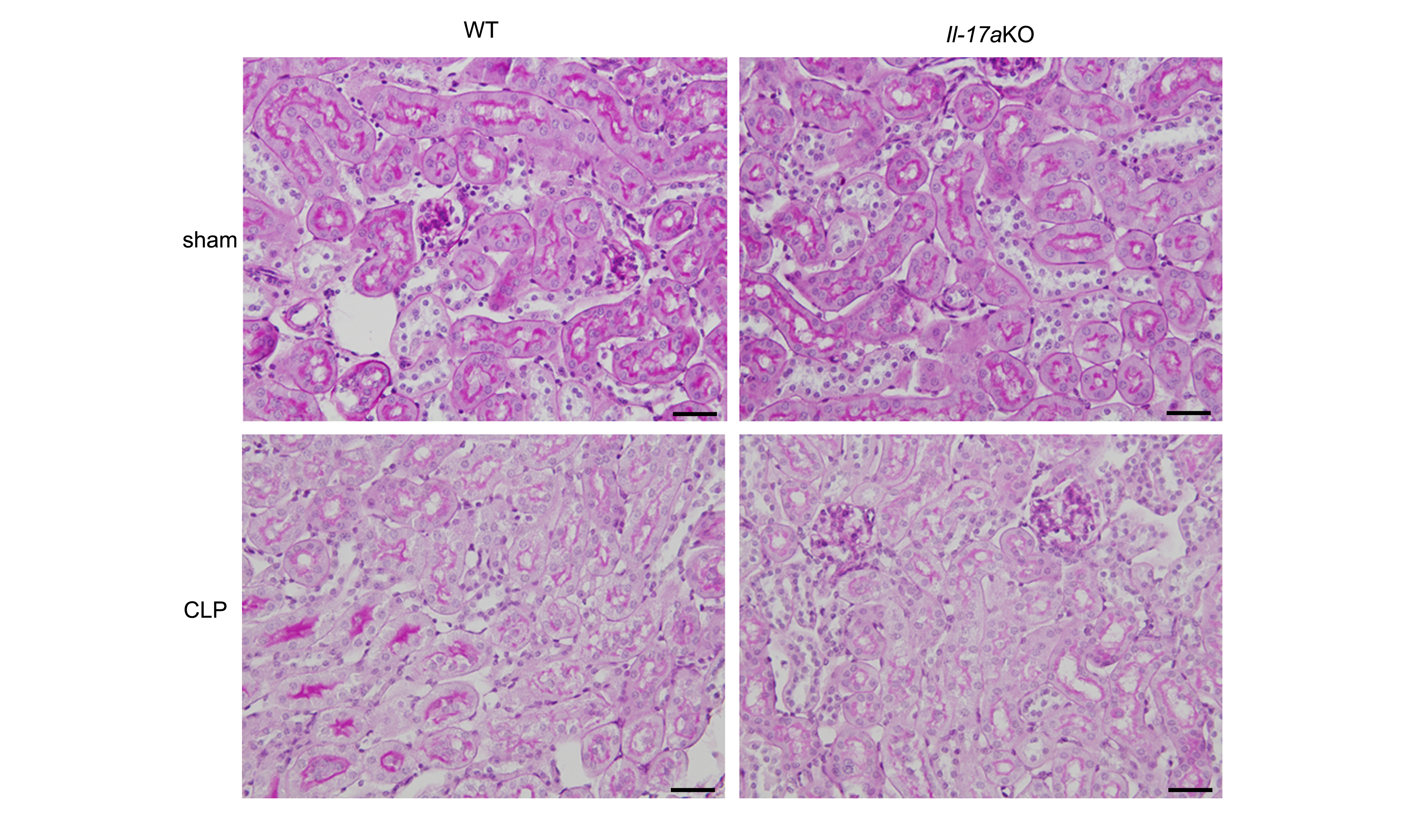

Supplement: Supplementary file 5 — Supplementary Material 5 [file 41598_2025_34770_MOESM5_ESM.jpg]

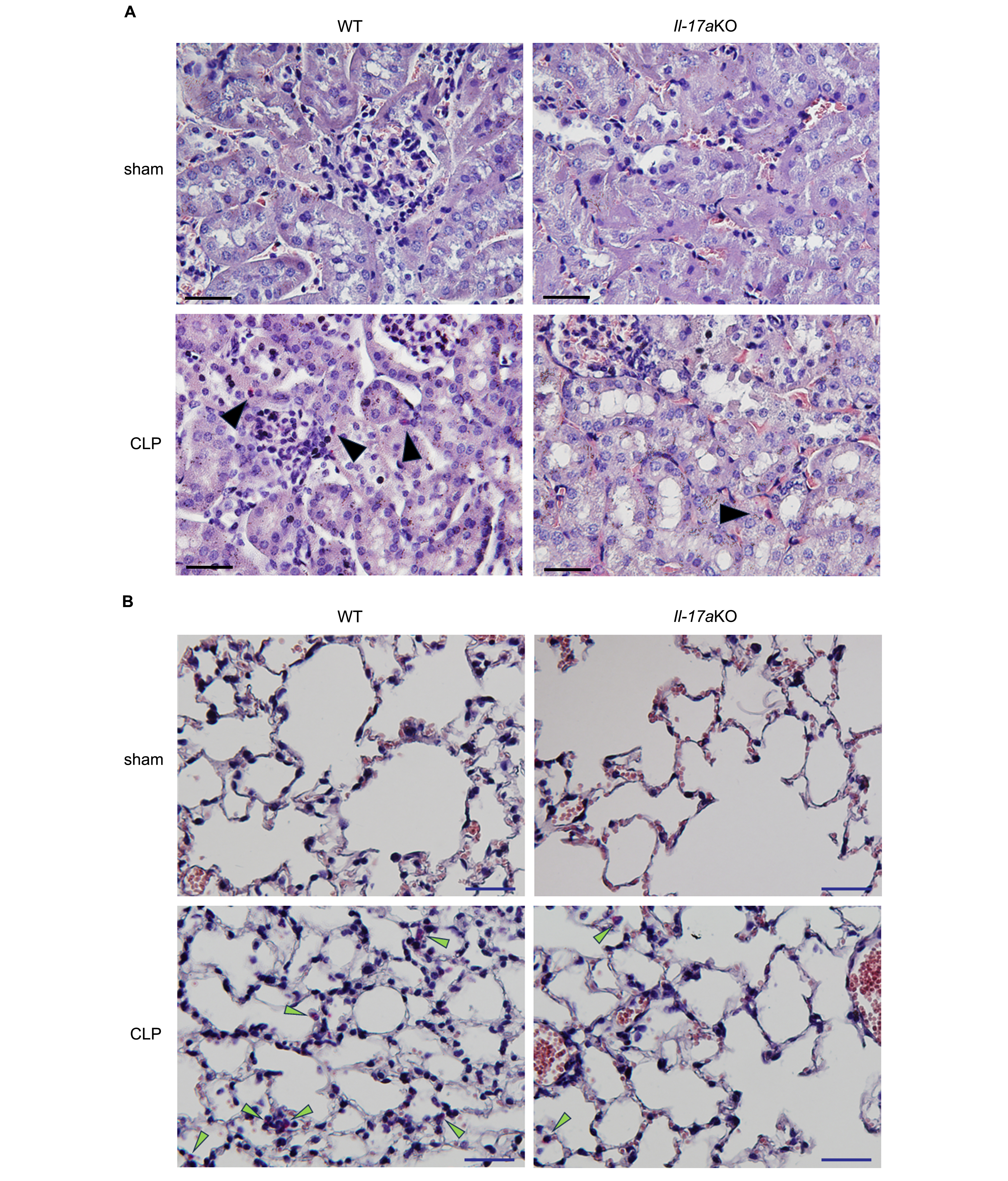

Supplement: Supplementary file 6 — Supplementary Material 6 [file 41598_2025_34770_MOESM6_ESM.jpg]

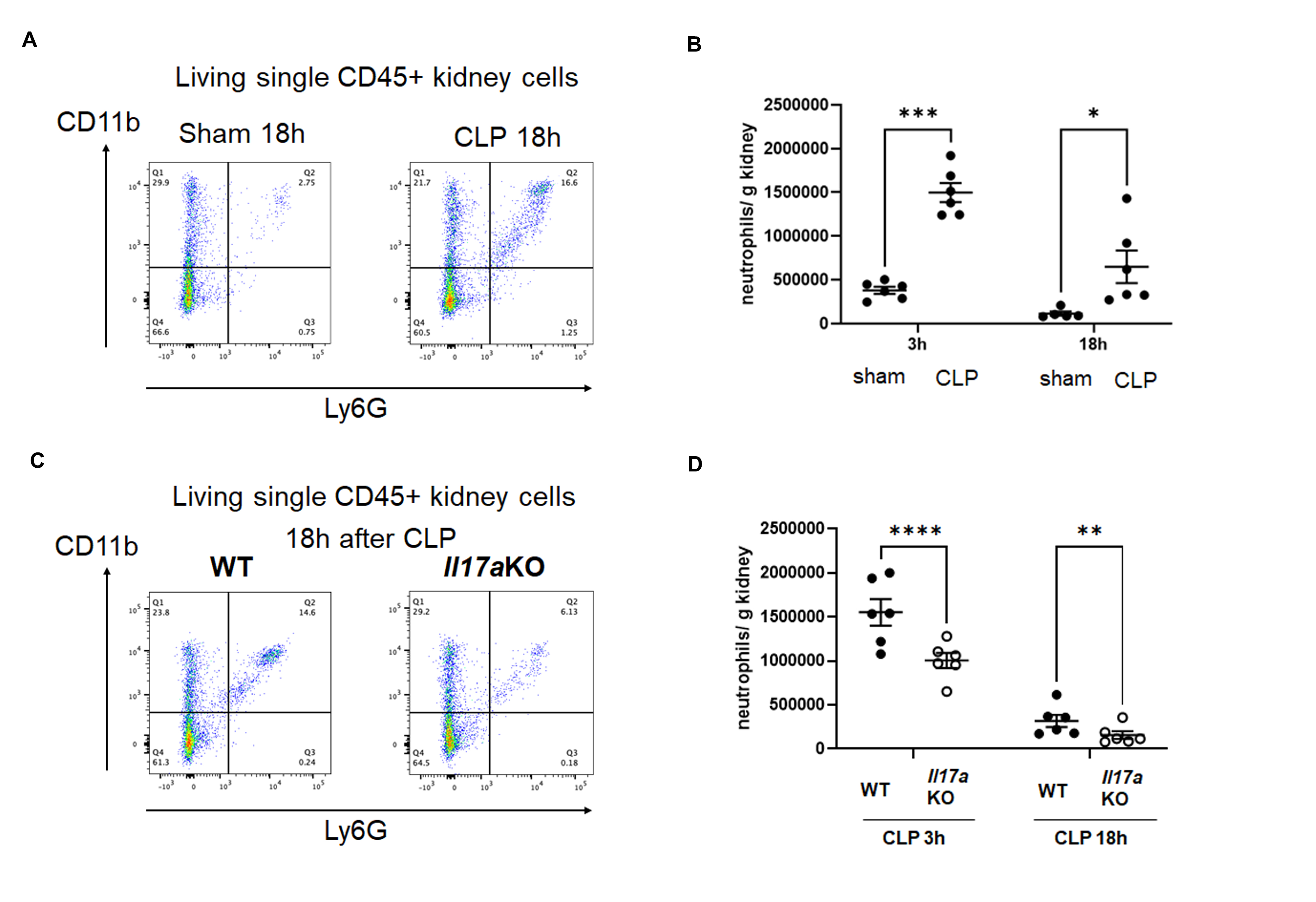

Supplement: Supplementary file 7 — Supplementary Material 7 [file 41598_2025_34770_MOESM7_ESM.jpg]

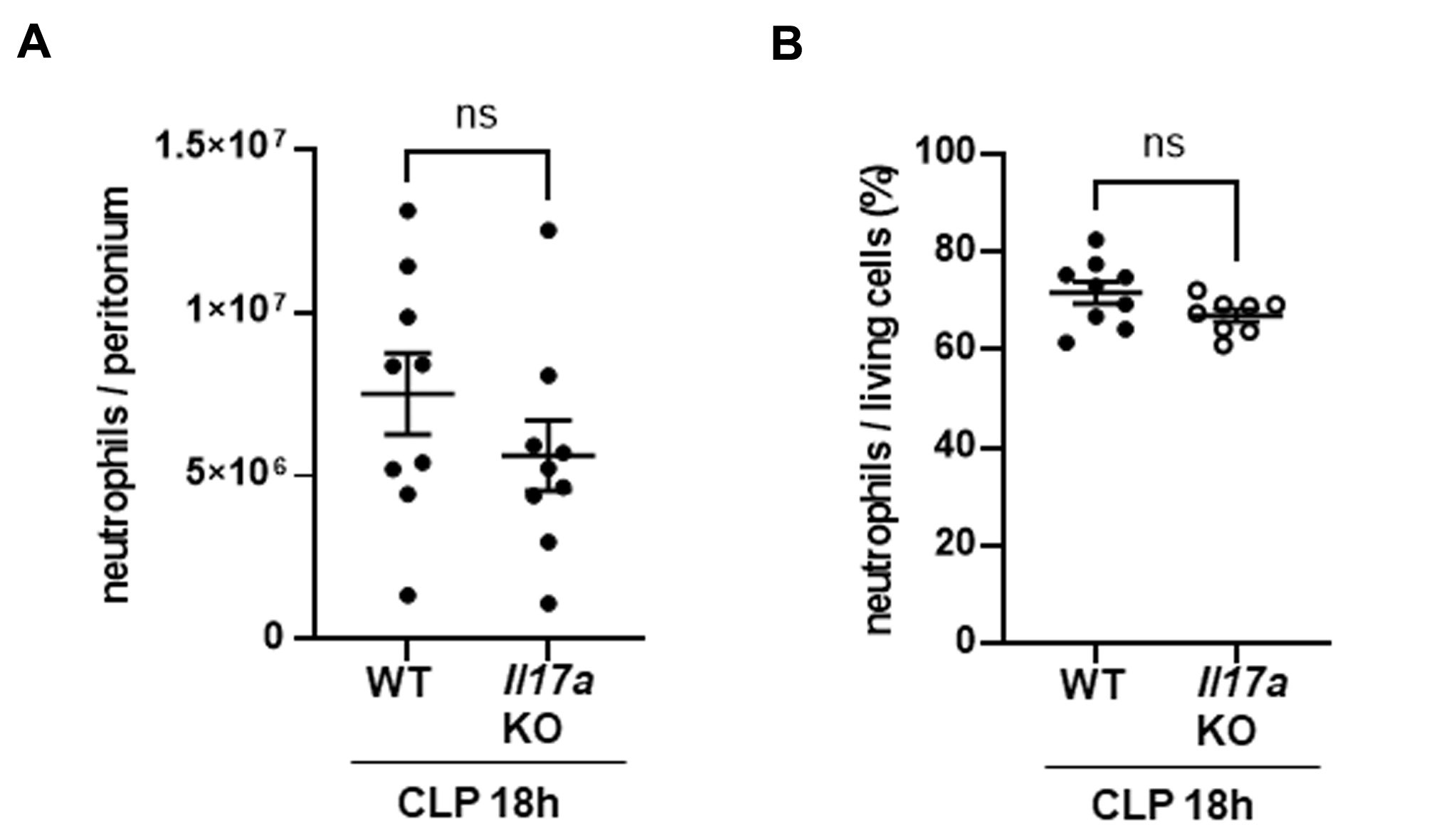

Supplement: Supplementary file 8 — Supplementary Material 8 [file 41598_2025_34770_MOESM8_ESM.jpg]

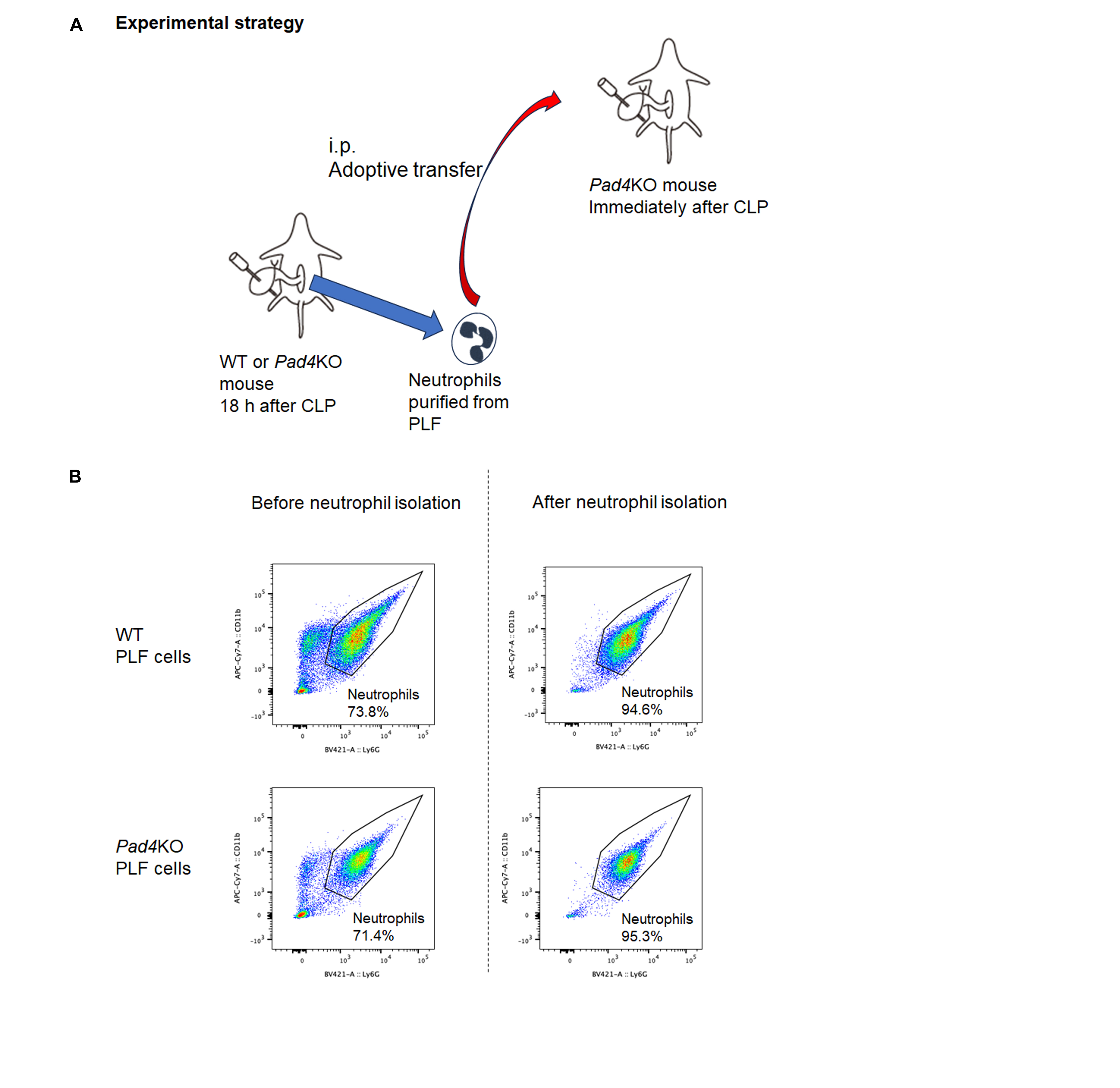

Supplement: Supplementary file 9 — Supplementary Material 9 [file 41598_2025_34770_MOESM9_ESM.jpg]

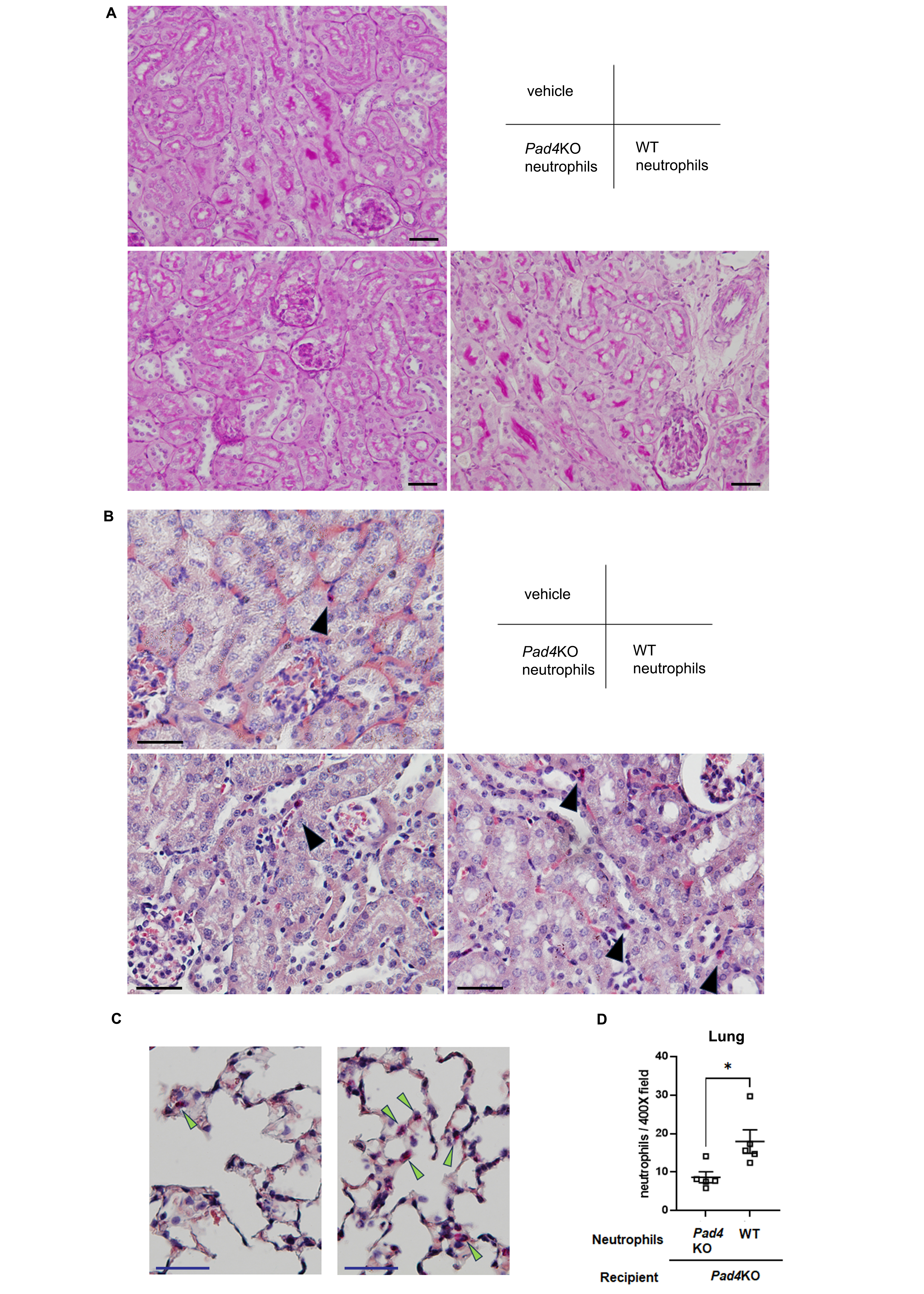

Supplement: Supplementary file 10 — Supplementary Material 10 [file 41598_2025_34770_MOESM10_ESM.jpg]

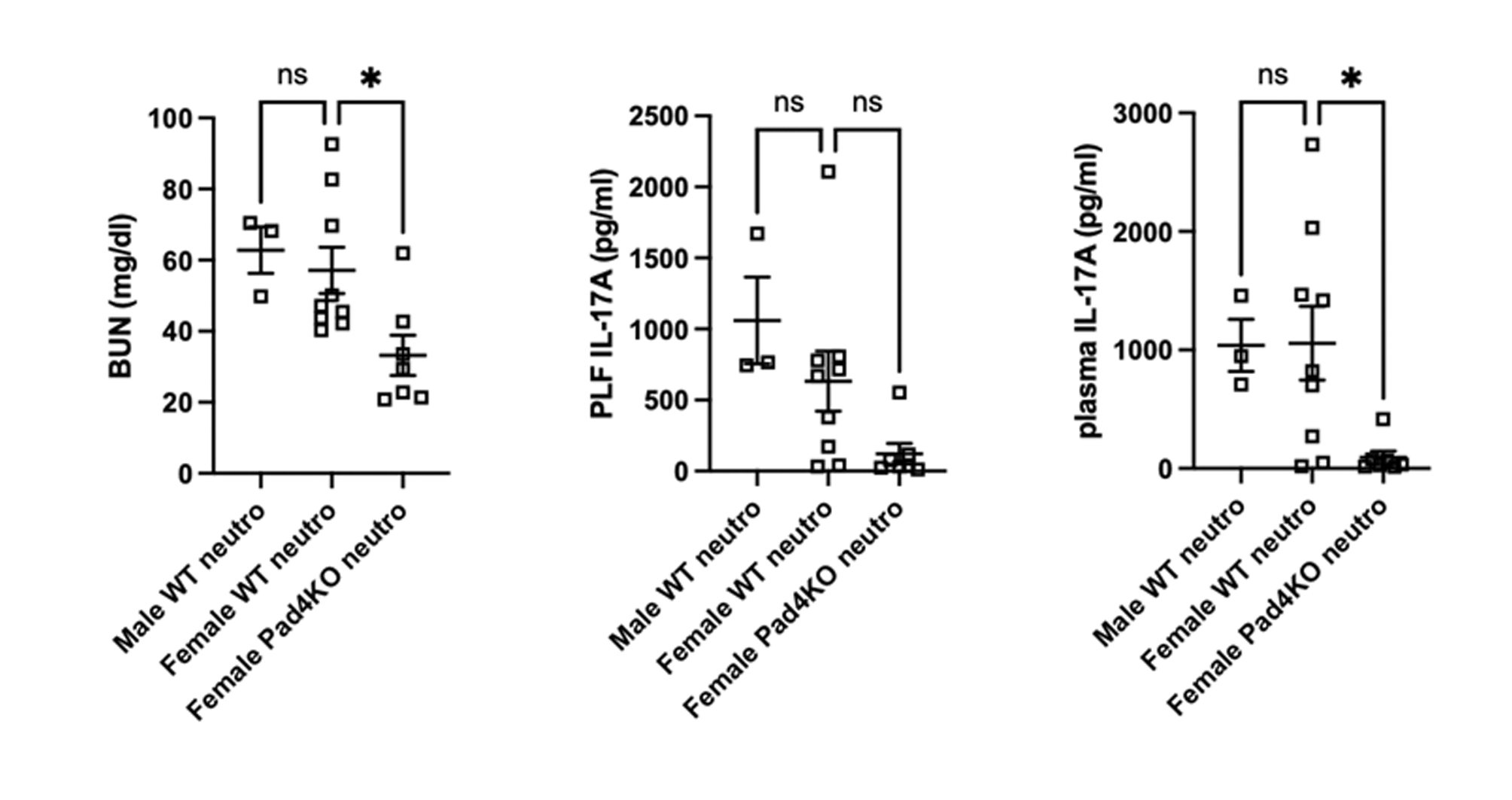

Supplement: Supplementary file 11 — Supplementary Material 11 [file 41598_2025_34770_MOESM11_ESM.jpg]
