# Supplementary material for: Peritoneal neutrophil extracellular traps contribute to septic AKI via peritoneal IL-17A and distant organ CXCL-1/ CXCL-2 pathway in abdominal sepsis
Source: Sci Rep. 2026 Jan 27;16:5446. doi: 10.1038/s41598-025-34770-1 (PMC12886817; doi:10.1038/s41598-025-34770-1)
Supplement: Supplementary file 12 — Supplementary Material 12 [file 41598_2025_34770_MOESM12_ESM.pdf]

Antibody	clone	catalog #	Vendeor	concentration(μg/ml)
APC Rat Anti-Mouse CD45	30-F11	103112	Biolegend	1.2
APC/Cy7 Armenian Hamster Anti-Mouse TCR	GL3	118143	Biolegend	1.2
APC/Cy7 Rat Anti-Mouse CD11b	M1/70	101226	Biolegend	1.2
FITC Rat Anti-Mouse F4/80	BM8	123108	Biolegend	5
FITC Mouse Anti-Mouse CD64	X54-5/7.1	139316	Biolegend	5
BV605 Rat Anti-Mouse Ly6G	1A8	127639	Biolegend	2
BV650 Rat Anti-Mouse CD8a	53-6.7	100741	Biolegend	0.5
BV786 Rat Anti-Mouse CD4	GK1.5	100453	Biolegend	2
PE/Cy7 Mouse Anti-Mouse NK-1.1	PK136	108714	Biolegend	4.8
PE Rat Anti-Mouse CD3	17A2	100206	Biolegend	8
Pacific Blue Rat Anti-Mouse Ly6G	1A8	127612	Biolegend	5
PE-CF594 Rat Anti-Mouse CD19	1D3	562291	BD	4
AF700 Rat Anti-Mouse CD3	17A2	100216	Biolegend	20
